# Observing Transient Breathing States of MIL‐53 Homologues Using In Situ Single Crystal 3D Electron Diffraction

**DOI:** 10.1002/smll.202509071

**Published:** 2025-10-30

**Authors:** Matthew Liddle, Celine Beck, Russell M. Main, Dominic Bara, Claire Wilson, Donald A. MacLaren, David Boldrin, Ross S. Forgan

**Affiliations:** ^1^ School of Chemistry University of Glasgow Glasgow G12 8QQ UK; ^2^ SUPA School of Physics and Astronomy University of Glasgow Glasgow G12 8QQ UK; ^3^ I11 High‐resolution Powder Diffraction Beamline Diamond Light Source Ltd. Harwell Science and Innovation Campus Didcot Oxfordshire OX11 0DE UK

**Keywords:** breathing, crystallography, electron diffraction, flexible, metal–organic frameworks

## Abstract

The MIL‐53 series of metal–organic frameworks (MOFs) is considered archetypal for flexible MOFs, with desolvated materials rapidly transitioning between open and closed phases in a stepwise breathing process in response to changes in temperature under ambient pressure conditions. Herein, the differing structures of MIL‐53(Cr) and MIL‐53(Ga) during breathing – hydrated and anhydrous, closed and open pore–are characterized by in situ single crystal 3D electron diffractionthrough varying sample conditions within the electron diffractometer. In doing so, the crystal structures of nine intermediate phases are uncovered that together represent the continuous breathing of MIL‐53 under vacuum, in stark contrast to ambient pressure stepwise breathing. In addition, these structures offer insight into particle‐to‐particle structural heterogeneity that are averaged out by conventional powder X‐ray diffraction measurements, and may begin to explain metal‐dependent adsorption phenomena observed across the MIL‐53 homologues. In situ 3D electron diffraction is therefore expected to become a powerful tool for in‐depth structural investigations of flexible porous materials.

## Graphical abstract

Temperature‐controlled in situ desolvation within the vacuum chamber of a dedicated electron diffractometer is shown to enable precise characterization of structural changes in flexible MOFs. Characterization of transient intermediate states of MIL‐53(Cr) and MIL‐53(Ga) at different levels of openness by 3D electron diffraction shows they undergo metal‐dependent, continuous breathing under vacuum, in contrast to stepwise breathing under ambient conditions.

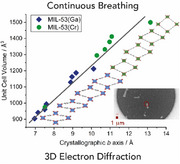

## 1 Introduction

Certain metal–organic frameworks (MOFs), coordination networks of metal ions or clusters connected by organic linkers into nets with potential porosity,^[^ ^1^ ^]^ can exhibit flexibility in response to external stimuli such as pressure, temperature, or guest exchange.^[^ ^2^, ^3^, ^4^, ^5^, ^6^, ^7^, ^8^ ^]^ The MIL‐53(M) series, with formula [M(OH)(BDC)]*_n_* (M = Sc^3+^, Cr^3+^, Fe^3+^, Al^3+^, Ga^3+^, In^3+^; BDC = benzene‐1,4‐dicarboxylate) can be considered archetypal for flexible MOFs.^[^ ^9^ ^]^ MIL‐53 was first isolated as the Cr homologue, MIL‐53(Cr),^[^ ^10^, ^11^ ^]^ comprising a 4^4^ net of BDC ligands connected by a 1D [Cr(OH)_2_(RCO_2_)_4_]*_n_* chain of bridging carboxylates and *trans* µ_2_‐OH units linking corner‐sharing Cr^3+^ octahedra into the **sra** topology. The MOF contracts and expands in a wine‐rack like motion by hingeing of the carboxylate units in response to temperature,^[^ ^10^, ^11^ ^]^ pressure^[^ ^12^ ^]^ and inclusion of guest molecules.^[^ ^13^, ^14^, ^15^, ^16^, ^17^, ^18^ ^]^ In the absence of guests at ambient pressure, MIL‐53(Cr) displays stepwise breathing behavior through discrete isolated states, in contrast to the continuous breathing exhibited by the MIL‐88(M) isoreticular series (M = similar trivalent metal cations),^[^ ^19^, ^20^ ^]^ where 6‐connected [M_3_O(RCO_2_)_6_(OH_2_)_2_(X)] (X = monoanion such as OH^−^ or F^−^) secondary building units are connected into hexagonal **acs** topology nets that display dramatic expansion of up to 270% in volume.^[^ ^21^, ^22^ ^]^

Flexible MOFs have been touted as potentially highly selective adsorbents for molecular storage, separation, and sensing.^[^ ^23^, ^24^ ^]^ Additionally, harnessing their pressure driven structural changes could lead to their application as multiferroics,^[^ ^25^ ^]^ shock absorbers,^[^ ^26^ ^]^ and barocalorics.^[^ ^27^ ^]^ Crystallographic characterization of structural changes is essential to understand and rationalise these behaviors, but the kinetic inertness of the Cr^3+^ ion limits crystal growth and has led, as far as we are aware, to no reports of a directly obtained single crystal structure of MIL‐53(Cr) to date; structural information around the varying phases that can be observed has come from powder X‐ray diffraction (PXRD). Literature nomenclature is inconsistent across different MIL‐53(M) homologues, complicating their comparison. In an attempt to remedy this, we have adopted a universal naming scheme (Table S1, Supporting Information) for this study. The closed pore (cp) monohydrate (mh) form, MIL‐53(Cr)_cp_mh (typically referred to as MIL‐53(Cr)_lt), is contracted and contains hydrogen‐bonded water molecules within its rhombic pores (unit cell volume ≈1000 Å^3^) with an overall composition of [Cr(OH)(BDC)]·H_2_O. On heating and resultant water desorption, the open pore (op) anhydrous (ah) form, MIL‐53(Cr)_op_ah (typically referred to as MIL‐53(Cr)_ht), is observed, with a dramatic increase in unit cell volume (*V* ≈ 1500 Å^3^) a consequence of pore expansion. Exclusion of water ensures activated MIL‐53(Cr) can adsorb N_2_ at 77 K, with BET areas variously reported in the region of 1300–1500 m^2^ g^−1^,^[^ ^28^, ^29^, ^30^ ^]^ whilst a “superhydrated” form (*V* ≈1550 Å^3^) has also been characterized by PXRD analysis of an aqueous paste.^[^ ^17^ ^]^

Homologues of MIL‐53(Cr) containing other metals, in particular MIL‐53(Ga), have been characterised by single crystal X‐ray diffraction,^[^ ^7^, ^31^, ^32^, ^33^, ^34^, ^35^ ^]^ but the low mechanical stability of single crystals of flexible MOFs under large amplitude volume change^[^ ^36^ ^]^ often precludes probing of breathing in single crystals, while comparisons across homologues are hindered by the fact that flexibility of MIL‐53 is metal dependent.^[^ ^9^ ^]^ For example, MIL‐53(Al) displays temperature‐dependent hysteresis in traversing its anhydrous closed and open pore forms,^[^ ^37^ ^]^ MIL‐53(Fe) adopts two narrow pore phases on dehydration and heating rather than an open pore phase,^[^ ^38^ ^]^ and MIL‐53(Sc) dehydrates to a further, very narrow pore form at elevated temperatures.^[^ ^39^ ^]^ Taken together, these factors led us to consider single crystal 3D electron diffraction (3D‐ED)^[^ ^40^, ^41^ ^]^ as an alternative methodology to obtain single crystal structures of MIL‐53(Cr), with the temperature‐controlled vacuum conditions within a dedicated electron diffractometer an ideal environment to probe structural flexibility in situ.^[^ ^42^, ^43^ ^]^ For example, we have observed in situ *c*‐axis contraction of a 2D metal–organic nanosheet material, GUF‐14(Zr), through desolvation under vacuum by 3D‐ED,^[^ ^44^ ^]^ whilst others have used variations in temperature to probe subtle structural changes in MOF‐74 materials as coordinated solvents are removed.^[^ ^45^ ^]^ A previous environmental transmission electron microscopy (ETEM) study, combined with molecular modeling, showed diffraction images consistent with the unit cells of MIL‐53(Cr)_op_ah at 300 K under vacuum and MIL‐53(Cr)_cp_mh at 300 K at 3 mbar water vapor pressure in the ETEM cell, but no crystal structures were solved.^[^ ^46^ ^]^ Most recently, 3D‐ED was used to determine the structures of microcrystals of MIL‐53(Al) in the as‐synthesised state (with unreacted BDC‐H_2_ in the pores), the closed pore monohydrate phase, and the open pore anhydrous phase, correlating closely with previous structures determined by powder and single crystal X‐ray diffraction.^[^ ^47^ ^]^ Herein, we show that, as well as obtaining single crystal structures of previously observed MIL‐53 phases, in situ 3D‐ED can be used to identify transient, metastable intermediate structures indicative of a continuous breathing process, in contrast to previous observations that breathing is stepwise under ambient pressure conditions. By examining both MIL‐53(Cr) and MIL‐53(Ga), we also show that metal‐dependent differences in breathing behavior can be investigated by 3D‐ED, complementing existing PXRD studies and potentially shedding light on contrasting adsorption properties across the MIL‐53(M) homologues.

## 2 Results and Discussion

**Figure 1:**
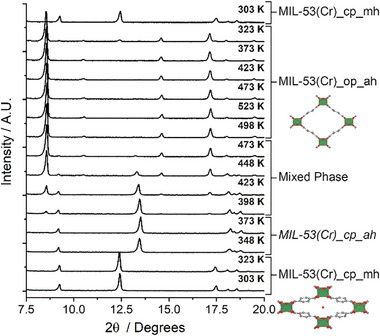
Stacked partial powder X‐ray diffractograms of MIL‐53(Cr) collected (from bottom to top) by heating MIL‐53(Cr)_cp_mh from 303 to 523 K then cooling back to 303 K. Annotations show phases present (confirmed by Le Bail fitting, Figures S4–S6, Supporting Information) with inset structural images of MIL‐53(Cr)_cp_mh (CSD code GUSNEN) ^[^ ^11^ ^]^ and MIL‐53(Cr)_op_ah (CSD code MINVUA)^[^ ^10^ ^]^ derived from PXRD and modeling. The previously unreported closed pore anhydrous phase, MIL‐53(Cr)_cp_ah, is observed at 348 and 373 K. Full diffractograms are found in the Figure S3 (Supporting Information).

Previous studies have shown the potential of electron diffraction to probe structural changes in flexible MOFs that cannot be obtained as large single crystals suitable for X‐ray diffraction.^[^ ^46^, ^47^ ^]^ We therefore sought to study MIL‐53(Cr), whose flexibility has been the subject of significantly less analysis than the other homologues in the MIL‐53 series, by 3D‐ED. MIL‐53(Cr) was prepared by our own, HF‐free aqueous route using HCl as modulator, and isolated as the closed pore hydrated form, MIL‐53(Cr)_cp_mh, by sequential activation in *N*,*N*‐dimethylformamide (DMF) and methanol.^[^ ^28^ ^]^ To benchmark the potential temperature regimes where structural transitions may be expected, MIL‐53(Cr)_cp_mh was first subjected to variable temperature PXRD analysis (**Figure** **1**). Surprisingly, heating from room temperature to 373 K resulted in the formation of an anhydrous, closed pore form of MIL‐53(Cr) that has not previously been reported. The diffractogram of MIL‐53(Cr)_cp_ah corresponds closely to the high temperature, anhydrous form of the iron homologue (previously denoted MIL‐53(Fe)_ht), which is found at temperatures over 423 K.^[^ ^38^ ^]^ This phase, with a unit cell volume ≈900 Å^3^ determined by Le Bail fitting, has also been observed for the Ga homologue (previously named MIL‐53(Ga)_lt)^[^ ^48^ ^]^ and the Al homologue (previously named MIL‐53(Al)LT).^[^ ^37^ ^]^ On heating to around 423 K, the structure began to open to the previously reported MIL‐53(Cr)_op_ah, with the transition completed on reaching 473 K. The stepwise nature of this transition under these conditions is evident in the diffractogram recorded at 423 K, which shows well‐resolved Bragg peaks for both phases. On cooling to 303 K, the structure returns to the MIL‐53(Cr)_cp_mh phase by readsorbing atmospheric moisture, as observed previously.^[^ ^11^ ^]^ These experiments confirm temperature‐dependent breathing of MIL‐53(Cr), but also suggest that additional phases could be observed and structurally characterized through careful control of the sample environment.

Samples of MIL‐53(Cr)_lt were deposited on holey carbon coated Cu TEM grids and cryogenically transferred to a Gatan ELSA cryoholder, prior to insertion into the Rigaku XtaLAB SynergyED electron diffractometer (Section S1, Supporting Information). The samples were held at 175 K, in order to preserve the hydration of the MOF in the vacuum chamber while removing by sublimation any residual ice crystals derived from atmospheric moisture during cryo‐transfer. It was possible to obtain two different single crystal structures of MIL‐53(Cr)_cp_mh with the pore‐bound water molecules clearly resolved from single crystal electron diffraction data collected at this temperature. Previous structural refinements from PXRD and ED data across homologues linked by Cr^3+^, Fe^3+^, Al^3+^, and Ga^3+^ have produced two possible solutions with general stoichiometry [M(OH)(BDC)]·H_2_O, which we term a monohydrate: a centred monoclinic structure (variously *C*2/*c* or *Cc*)^[^ ^11^, ^49^, ^50^ ^]^ and a primitive monoclinic structure (space group 14, *P*2_1_/*c* or *P*2_1_/*n* settings) with a doubling of the unique monoclinic *b*‐axis.^[^ ^32^, ^38^, ^48^, ^51^ ^]^ Intermediate solutions with partially occupied, disordered water molecules have also been reported (see Table S2, Supporting Information).^[^ ^17^, ^52^ ^]^ These inconsistent solutions led to a recent study of MIL‐53(Al) by terahertz spectroscopy, where the spectroscopic data agreed closely with the structure of the monohydrate modeled from the primitive monoclinic structure.^[^ ^53^ ^]^ The previously reported structures of MIL‐53(Cr)_cp_mh, derived from PXRD, are both *C*‐centered monoclinic.^[^ ^11^, ^17^ ^]^

**Figure 2:**
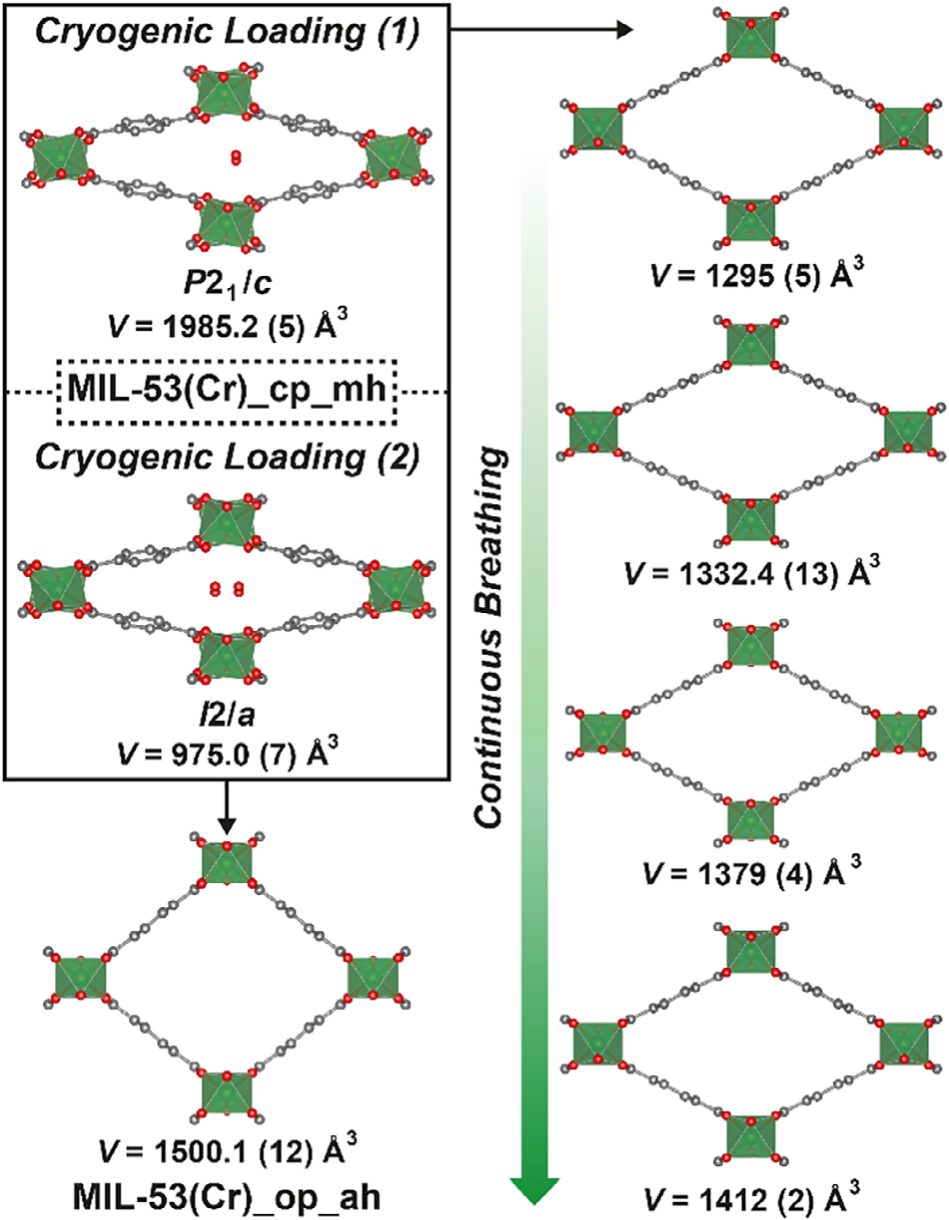
Single crystal structures of MIL‐53(Cr) in various states obtained by electron diffraction, annotated with unit cell volumes and space groups as appropriate. Arrows indicate sample treatment within the diffractometer: the transient intermediate structures (right) were derived from the sample grid that gave the primitive monoclinic structure of MIL‐53(Cr)_cp_mh, whilst MIL‐53(Cr)_op_ah (bottom left) was obtained from the sample grid that gave the *I*‐centered monoclinic structure of MIL‐53(Cr)_cp_mh. H atoms removed for clarity. C: gray; O: red; Cr: green polyhedra.

3D‐ED data were collected for two crystal structures of MIL‐53(Cr)_cp_mh (**Figure** **2**). In our case, the data for the two forms were collected from individual crystals on separate cryogenically loaded sample grids, but both forms are likely to have been present on each sample grid, as the same synthetic sample of MIL‐53(Cr)_cp_mh was used each time. One structure solves in the primitive monoclinic space group *P*2_1_/*c* (no. 14, *V* = 1985.2(5) Å^3^) and unambiguously shows two symmetry independent, fully occupied water molecules corresponding to the monohydrate, as there are two symmetry independent Cr sites and two BDC linkers, (one complete and two half BDC linkers) in the asymmetric unit. This structure aligns with a recent X‐ray crystal structure of the Ga^3+^ homologue^[^ ^32^ ^]^ and the 3D‐ED structure of the Al^3+^ homologue.^[^ ^47^ ^]^ The second structure solves in the *I*2/a space group (no. 15, *V* = 975.0(7) Å^3^), and is consistent with the previous^[^ ^11^, ^17^ ^]^ *C*‐centred monoclinic structures (*I*2/*a* and *C*2/*c* are different settings of space group no. 15), with a single, half‐occupied independent water molecule per asymmetric unit cell, disordered over two symmetry related positions (the asymmetric unit additionally contains 0.5[Cr(OH)(BDC)]). However, the high atomic displacement parameters (adps) of the half‐occupied O atom of the water molecule in the *I*2/a structure indicated a potential substoichiometric hydrate; the occupancy was freely refined to ≈0.43(3), giving a formula of [Cr(OH)(BDC)]·0.86H_2_O. We hypothesize, therefore, that partial dehydration of MIL‐53(Cr)_cp_mh may lead to subtle structural reorganization of the pore‐bound water molecules and could explain the varying structural solutions of the closed pore, approximately monohydrated MIL‐53 materials.

Heating the samples to 300 K whilst still under vacuum resulted in dehydration, allowing determination of the crystal structure of MIL‐53(Cr)_op_ah. The unit cell volume of 1500.1(12) Å^3^ (space group no. 74, *Imma*) corresponds very closely to the original PXRD analyses of MIL‐53(Cr)_op_ah (*V* = 1486.14 Å^3^),^[^ ^10^, ^11^ ^]^ and the single crystal structures of MIL‐53(Ga) _op_ah (X‐ray diffraction, *V* = 1501.1(3) Å^3^)^[^ ^32^ ^]^ and MIL‐53(Al) _op_ah (electron diffraction, *V* = 1460.63 Å^3^),^[^ ^47^ ^]^ both generated by in situ heating (see Table S3, Supporting Information). Whilst under ambient pressure conditions, generation of this open pore phase typically requires high temperatures, it is clear that a high vacuum environment is sufficient to drive off bound water and open the structure at room temperature; our observations correlate with the previous ETEM work on MIL‐53(Cr) at 300 K^[^ ^46^ ^]^ and the 3D‐ED structure of MIL‐53(Al) recorded at 293 K,^[^ ^47^ ^]^ both run under vacuum. In addition, simulations on the energy landscape of MIL‐53(Cr) predict that, at 293 K, decreasing pressure energetically favors the open pore phase compared to the closed pore phase.^[^ ^54^ ^]^

In addition to MIL‐53(Cr)_op_ah, four further structures with intermediate unit cell volumes, from 1295(5) – 1412(2) Å^3^, were collected across different temperatures, unexpectedly indicating continuous breathing of the individual MIL‐53(Cr) microcrystals rather than the stepwise structural change observed by PXRD. Despite cooling the dehydrated sample back to 100 K, the anhydrous closed pore phase (*V* ≈ 900 Å^3^) was not observed. These additional, intermediate structures have only been observed under vacuum and in the temperature range 100 – 327 K, and clearly show the transient, metastable conformations through which anhydrous MIL‐53(Cr) must travel as its pores open and close. Simulations carried out at 0 K predict the closed pore form to be energetically favorable at this temperature, compared to the open pore being favorable at 293 K,^[^ ^54^ ^]^ correlating with the gradual closing that is observed. We believe that the closed pore anhydrous form has not been observed in these experiments as the sample cannot be cooled to a temperature low enough to induce it. Compared to ambient pressure measurements, we observe MIL‐53(Cr)_op_ah at much lower temperatures under vacuum, suggesting the entire temperature‐dependent breathing behavior of MIL‐53(Cr) is shifted to a much lower temperature regime when under vacuum compared to ambient pressure. It is clear from these experiments that the energetic landscape corresponding to the breathing of anhydrous MIL‐53(Cr) is complex and temperature dependent, but the high vacuum environment allows trapping of these metastable states.

**Figure 3:**
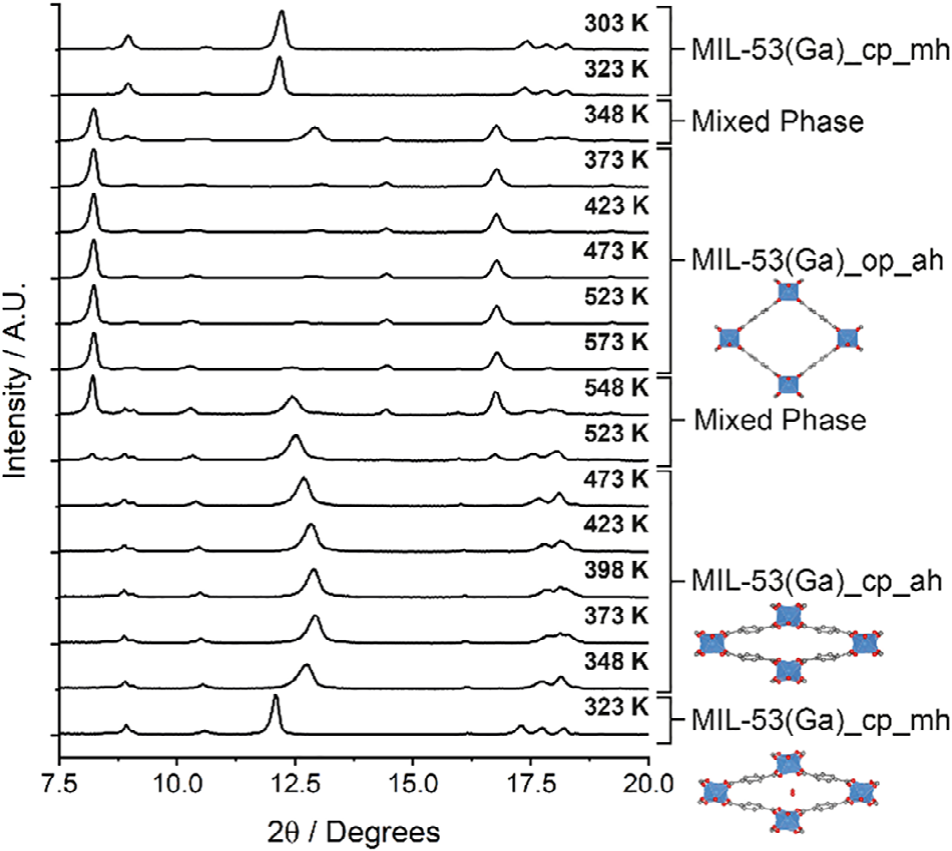
Stacked partial powder X‐ray diffractograms of MIL‐53(Ga) collected (from bottom to top) by heating MIL‐53(Ga)_cp_mh from 323 to 573 K then cooling back to 303 K. Annotations show phases present (confirmed by Le Bail fitting, Figures S8–S10, Supporting Information) with inset structural images of MIL‐53(Ga)_cp_mh (QOVWOO02), MIL‐53(Ga)_cp_ah (LOQLIN12), and MIL‐53(Ga)_op_ah (QOVWUU01).^[^ ^32^ ^]^ Full diffractograms are found in the Figure S7 (Supporting Information).

To determine if the ability to isolate these intermediate phases is metal‐dependent, MIL‐53(Ga) was prepared by solvothermal synthesis in DMF. After activation at 593 K under vacuum and exposure to the ambient atmosphere, it was isolated as MIL‐53(Ga)_cp_mh, as was observed for MIL‐53(Cr). Variable temperature PXRD analysis of MIL‐53(Ga)_cp_mh (**Figure** **3**) showed it exhibits similar breathing behavior to MIL‐53(Cr), albeit with phase transitions occurring at different temperature regimes, with formation of the closed pore anhydrous phase (previously denoted as MIL‐53(Ga)_lt) at around 348 K and the open pore anhydrous phase (previously denoted as MIL‐53(Ga)_ht) at around 473 K, closely correlating with previous work.^[^ ^48^ ^]^ Selected diffractograms underwent Le Bail fitting (see Figures S8–S10, Supporting Information) that confirmed formation of these phases, although the unit cell volumes suggested the MIL‐53(Ga)_cp_ah phase had not fully closed during the variable temperature run, whilst the Bragg peak at 2*θ* ≈ 12.5° for this phase shifted with temperature, suggesting minor additional breathing behavior.

**Figure 4:**
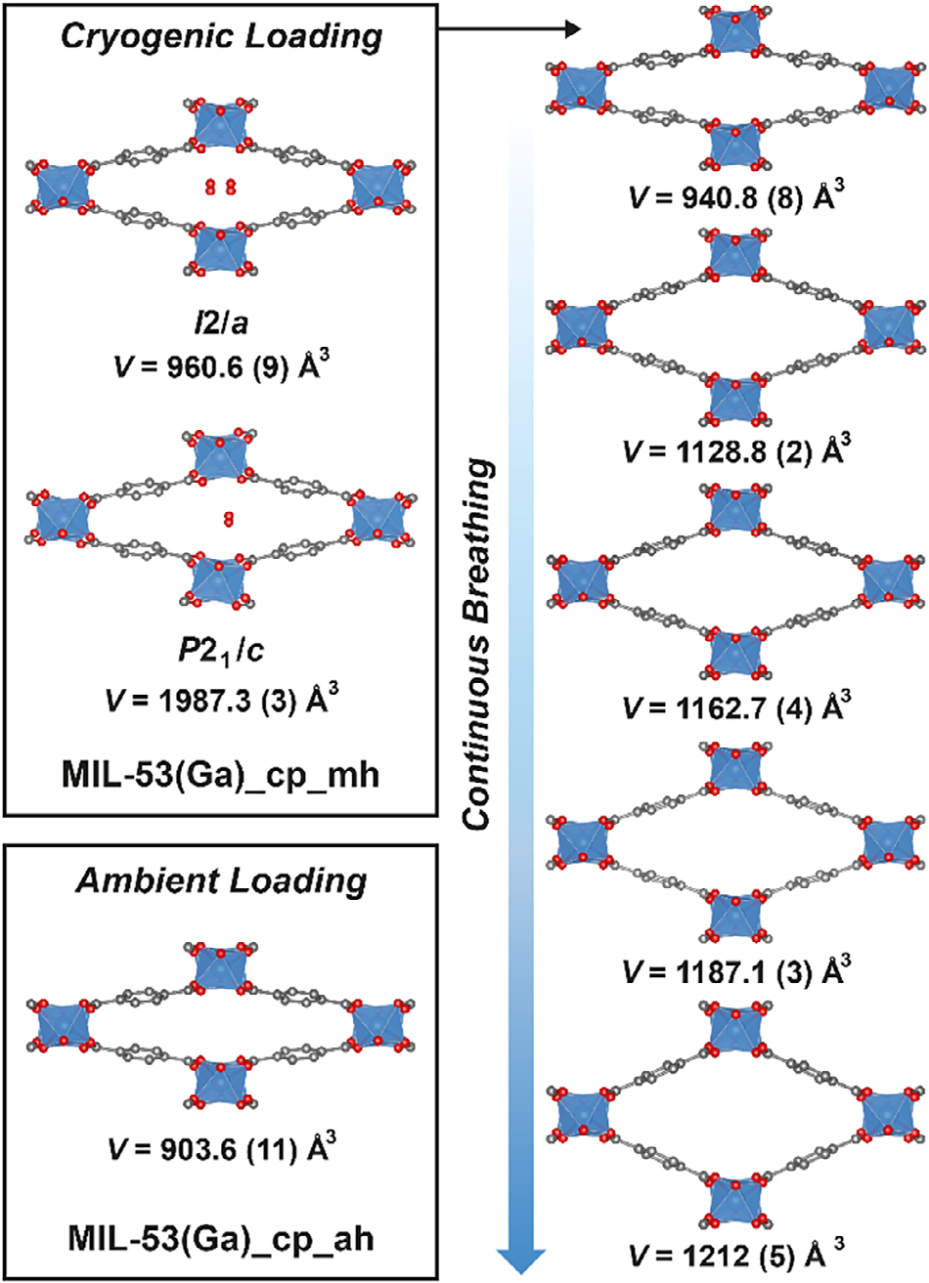
Single crystal structures of MIL‐53(Ga) in various states obtained by electron diffraction, annotated with unit cell volumes and space groups as appropriate. The arrow indicates sample treatment: all transient structures were derived from the grid that first yielded the structures of MIL‐53(Ga)_cp_mh. Possible partially occupied water molecules and H atoms removed for clarity. C: gray; O: red; Ga: blue polyhedra.

Flash freezing and cryogenic transfer also proved to be an expedient route to preserve the hydration of MIL‐53(Ga)_cp_mh for 3D‐ED experiments. As with MIL‐53(Cr), it was possible to obtain single crystal structures of both the primitive and centred monoclinic closed pore monohydrates of MIL‐53(Ga), in this case directly from individual crystals on the same sample grid (**Figure** **4**). As with the Cr homologues, the former solved in the primitive monoclinic *P*2_1_/*c* space group (no 14, *V* = 1987.3 (3) Å^3^) and the latter in the centred *I*2/a space group (no 15, *V* = 960.6(9) Å^3^); the monohydrated Ga and Cr homologues are essentially isostructural (Figure S11, Table S2, Supporting Information). The only notable difference is the *I*‐centered monoclinic structure with Ga has a slightly smaller unit cell volume than that with Cr, and the half‐occupied O of the pore‐bound disordered water refined fully to 0.5, corresponding to a monohydrate. The ability to obtain multiple datasets from individual crystals across one sample grid in this manner means that electron diffraction can reveal subtle particle heterogeneity that would be averaged by powder X‐ray diffraction.

The need for cryogenic loading and low temperature measurements to preserve hydration was demonstrated by the solution of the structure of MIL‐53(Ga)_cp_ah from a second grid loaded under ambient conditions and analysed at 298 K. The structure obtained by electron diffraction (*I*2/a, *V* = 903.6(11) Å^3^) correlates closely with that obtained by single crystal X‐ray diffraction (*I*2/a, *V* = 895.5(3) Å^3^) of a dehydrated sample (see Table S4, Supporting Information).^[^ ^32^ ^]^ In fact, the original cryogenically loaded sample was observed to dehydrate even when held at 175 K (i.e., below the temperature at which ice sublimes under vacuum). A structure was obtained in an intermediate open state (*I*2/a, *V* = 1128.8(2) Å^3^) with a residual peak in the difference potential map that could be modeled as a twofold positionally disordered water with an occupancy of 0.25; this would correspond to a hemihydrate, [Ga(OH)(BDC)]·0.5H_2_O. This potential partial hydrate with an increased unit cell volume shows the role of H‐bonded pore‐bound water in maintaining the closed pore state, and suggests that individual grains may be found in states, such as this unexpected partial hydrate, which are not observed across bulk measurements. The grid was subsequently held at 327 K to remove pore bound solvent and reanalysed, resulting in the observation of additional intermediate structures of MIL‐53(Ga) across different crystals, with unit cell volumes in the range 940.8(8) – 1212(5) Å^3^, proving that the ability to isolate transient intermediates in the breathing process was not limited to MIL‐53(Cr). Whilst it was not possible to establish a strong correlation between temperature and breathing of the transient intermediate states within the operating temperatures of the sample holder inside the electron diffractometer (100–327 K), which we believe is due to the influence of particle size on flexibility,^[^ ^55^, ^56^ ^]^ other than the closed pore monohydrates, the obtained structures for MIL‐53(Cr) all have larger unit cell volumes and are more “open” than those for MIL‐53(Ga) (all structures are collated in Table S5, Supporting Information). This aligns with the variable temperature PXRD analysis that shows higher temperatures are required under ambient conditions to access the open pore anhydrous form of MIL‐53(Ga) compared to MIL‐53(Cr). The closed pore anhydrous form of both can be observed by PXRD at 373 K at ambient pressure, yet we have not to date obtained the crystal structure of MIL‐53(Cr)_cp_ah by electron diffraction, suggesting more complex, metal‐dependent behavior under vacuum conditions.

The MIL‐53(Ga) structure with *V* = 940.8(8) Å^3^ is only slightly more open than MIL‐53(Ga)_cp_ah, and contains no pore‐bound water. The other structures have unit cell volumes in the range 1128.8(2)–1212(5) Å^3^ and exhibit residual potential peaks that could correspond to pore bound water with occupancies from 0.1–0.2, despite the sample having been heated at 327 K under vacuum. There is no correlation between the size of the residual peaks in the difference potential and the unit cell volume, so we are hesitant to ascribe them to pore‐bound solvent. Partial solvent loss would also not be expected to lead to more open phases; previous studies on solvent induced breathing require additional solvent to maintain partially open pore structures.^[^ ^17^, ^32^, ^57^ ^]^

**Figure 5:**
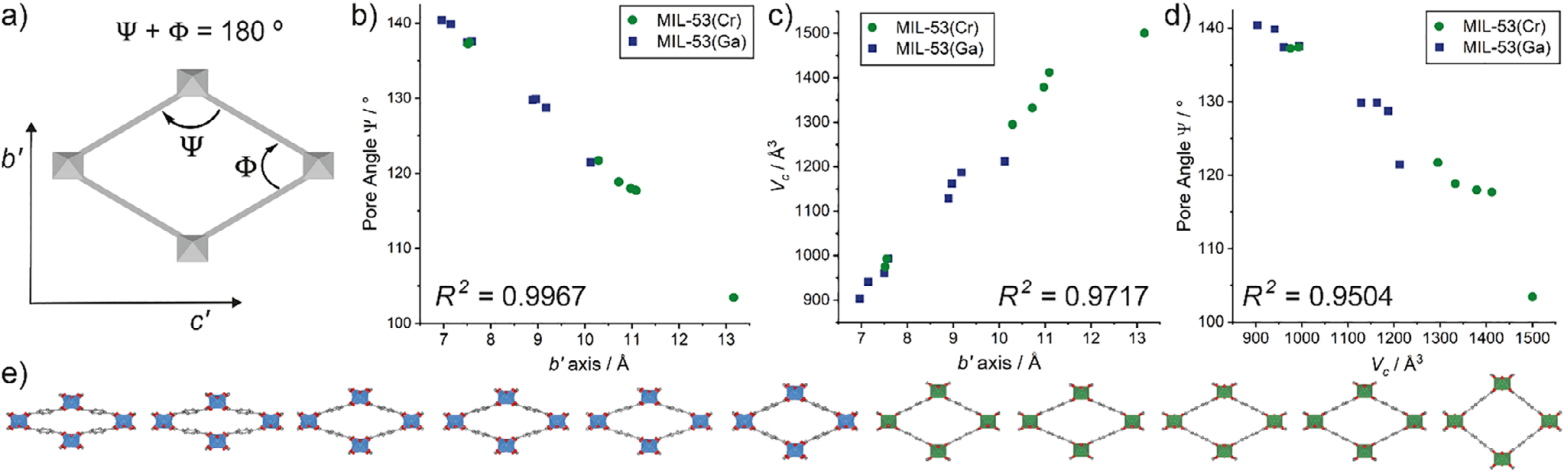
a) Schematic to illustrate universal unit cell parameters and angles. Plots of b) Pore angle Ψ versus universal *b′* axis, c) normalised pore volume *V*_c_ versus universal *b′* axis, and d) pore angle Ψ versus normalised pore volume *V*_c_ for all structures of MIL‐53(Cr) and MIL‐53(Ga) obtained by electron diffraction. Annotated with *R* ^2^ values for linear fits (Figures S12–S14, Supporting Information). e) Continuous breathing illustrated by plotting of all MIL‐53(Cr) and MIL‐53(Ga) crystal structures (not including hydrates) in order of Vc. Possible partially occupied water molecules and H atoms removed for clarity. C: gray; O: red; Cr: green polyhedra; Ga: blue polyhedra. Not to scale.

The varying structures obtained for MIL‐53(Cr) and MIL‐53(Ga) were analyzed to assess if the observed variations were indeed due to continuous breathing. The level of “open‐ness” of the structure was quantified by measuring the angles Ψ and φ (**Figure** **5a**) that correspond to the internal vertices of the rhombus shaped pore; a perfect square (Ψ = φ = 90) would be the most open conformation possible (the “superhydrated” MIL‐53(Cr) has φ = 88.6°, to the best of our knowledge the most open form reported to date^[^ ^17^ ^]^). To allow comparison of structures solved in different space groups, a normalized unit volume (*V*_c_) was defined containing four formula units, i.e., 4([M(OH)(BDC)]·xH_2_O); this was achieved by dividing the primitive monoclinic unit cell volumes by two. Universal unit cell parameters (*a′*, *b′*, and *c′*) were used (tabulated in Table S6, Supporting Information), where a′ runs parallel to the 1‐D chain SBU, whilst *b′* and *c′* lie orthogonal to the pore direction and are analogous to the cluster‐to‐cluster distances. The universal *b*‐axis parameter (*b′*) increases as the pore opens, and was also normalized where required by halving the primitive monoclinic unique *b*‐axis length.

2D plots of Ψ, *b'*, and *V*_c_ against one another reveal strong linear correlations (Figure 5b–d), confirming the continuous nature of the breathing observed across the transient, intermediate MIL‐53(M) crystal structures. The plots involving *V*_c_ deviate somewhat from linearity compared to the plot of Ψ versus *b'*, as represented in the *R^2^* values from the linear fits (see Figures S12–S14, Supporting Information), which may be a consequence of the fact that measurements were taken across different temperatures, leading to variations in the *a′*‐axis parameter (corresponding to the distances along the infinite chain SBU) and *β*′ angle, particularly for MIL‐53(Ga), and thus the overall unit cell volume. Nevertheless, the values confirm continuous breathing in MIL‐53(M) under vacuum (Figure 5e), in contrast to the stepwise breathing observed under ambient conditions.

## 3 Conclusion

This study has shown that both MIL‐53(Cr) and MIL‐53(Ga) exhibit continuous breathing under vacuum, with a number of transient intermediate structures observed by 3D‐ED that cannot be isolated under ambient pressure conditions. The work showcases 3D‐ED as a valuable tool to observe structural changes in flexible MOFs at the single crystal level, with stimuli‐responsive behavior possible in sample chambers wherein environmental conditions (e.g., temperature) can be modified. Many flexible MOF crystals crack or fracture upon desolvation, making it challenging to obtain their structures using single crystal X‐ray diffraction, but the lower particle size limit of electron diffraction (coupled with the high vacuum sample chamber) means structural characterization of desolvated, “closed” forms of flexible MOFs should become commonplace. In addition, cryogenic loading and environmental cells are likely to allow a range of complementary, solvated structures to be obtained.

By carefully desolvating crystals of MIL‐53(Cr) and MIL‐53(Ga) in situ, we have unveiled the structures of transient, metastable intermediate states that are not observed under ambient conditions, and are consistent with a continuous pathway between the anhydrous closed and open pore forms in these two MOFs (**Table** **1**). Our evidence also suggests the metal‐dependent breathing behavior that occurs under ambient conditions is reflected in the structures collected within the vacuum environment of the electron diffractometer; we are currently investigating this across other MIL‐53(M) homologues.

**Table 1:** Normalised unit cell data for all literature MIL‐53(Cr) and MIL‐53(Ga) structures and those from electron diffraction data in this study, listed in order of corrected unit cell volume *V*_c_.

X	SG^a^ ^)^	*a'*/Å^b^ ^)^	*b*'/Å^b)^, ^c)^	*c*'/Å^b^ ^)^	β*/*°	*V*/Å^3^	*V*_c_/Å^3^ ^d)^	refs.
Ga^e^ ^)^	*Cc* (9)	19.7749(2)	6.96751(8)	6.70007(8)	103.943(1)	895.95(2)	895.95(2)	[50]
Ga^f^ ^)^	*I*2/*a* (15)	6.7061(8)	6.914(2)	19.322(2)	95.68(1)	891.5(3)	891.5(3)	[32]
Ga	*I*2/a (15)	6.7449(19)	6.959(8)	19.343(6)	95.57(3)	903.6(11)	903.6(11)	This work
Ga	*I*2/a (15)	6.7570(12)	7.145(6)	19.569(3)	95.266(13)	940.8(8)	940.8(8)	This work
Ga^g^ ^)^	*P*2_1_/*c* (14)	6.680(1)	14.8550(7)	19.269(2)	96.224(3)	1900.8(3)	950.5	[32]
Ga	*I*2/a (15)	6.6980(18)	7.498(6)	19.239(5)	96.20(3)	960.6(9)	960.6(9)	This work
Ga^h^ ^)^	*P*2_1_/*c* (14)	19.7053(2)	15.1642(4)	6.68117(9)	103.7936(8)	1938.56(7)	969.3	[48]
Ga^h^ ^)^	*P*2_1_/*c* (14)	19.6801(2)	15.1165(1)	6.6716(1)	103.7573(9)	1927.83(3)	963.9	[51]
Ga^h^ ^)^	*Cc* (9)	19.6597(2)	7.6444(1)	6.6716(1)	103.8831(8)	973.36(2)	973.4	[50]
Cr	*I*2/a (15)	6.7892(16)	7.517(5)	19.202(4)	95.80(2)	975.0(7)	975.0(7)	This work
Cr	*P*2_1_/c (14)	6.8355(10)	7.5515	19.3338(14)	96.039(11)	1985.2(5)	992.6	This work
Ga	*P*2_1_/c (14)	6.7487(3)	7.5908	19.5011(10)	95.922(5)	1987.3(3)	993.7	This work
Cr^h^ ^)^	*C*2/*c* (15)	20.9168(2)	7.7005(1)	6.7780(2)	114.362(1)	994.521	994.5	[17]
Cr^h^ ^)^	*C*2/*c* (15)	19.685(4)	7.849(1)	6.782(1)	104.90(1)	1012.64	1012.6	[11]
Ga	*I*2/a (15)	6.6980(6)	8.8946(13)	18.9895(14)	93.837(6)	1128.8(2)	1128.8(2)	This work
Ga	*I*2/a (15)	6.7860(7)	8.962(3)	19.167(2)	94.111(10)	1162.7(4)	1162.7(4)	This work
Ga	*I*2/a (15)	6.7672(7)	9.179(2)	19.1391(18)	93.136(8)	1187.1(3)	1187.1(3)	This work
Ga	*I*2/a (15)	6.632(5)	10.12(2)	18.07(6)	92.31(11)	1212(5)	1212(5)	This work
Cr	*Imma* (74)	6.821(3)	10.29(3)	18.449(19)	90	1295(5)	1295(5)	This work
Cr	*Imma* (74)	6.8514(12)	10.719(9)	18.143(9)	90	1322.4(13)	1322.4(13)	This work
Cr	*Imma* (74)	6.890(4)	10.97(3)	18.254(14)	90	1379(4)	1379(4)	This work
Cr	*Imma* (74)	6.941(2)	11.088(17)	18.347(11)	90	1412(2)	1412(2)	This work
Ga^h^ ^)^	*Imma* (74)	6.7166	16.6784	13.2093	90	1479.7	1479.7	[48]
Cr^h^ ^)^	*Imcm* (74)	6.812(1)	16.733(1)	13.038(1)	90	1486.14	1486.14	[11]
Cr	*Imma* (74)	6.8369(12)	13.159(9)	16.674(6)	90	1500.1(12)	1500.1(12)	This work
Ga^i^ ^)^	*Imma* (74)	6.7401(7)	16.7753(17)	13.2673(18)	90	1500.1(3)	1500.1(3)	[32]

^a)^ SG = Space Group setting and number;

^b)^ Some space groups have had *a*, *b* and *c* reordered to allow comparison between unit cells;

^c)^ *P*‐centred *b* values have been halved to allow comparison with *C*‐ and *I*‐centred unit cells;

^d)^ *P*‐centred unit cell volumes halved to allow comparison with *C*‐ and *I*‐centred unit cells;

^e)^ Unit cell parameters obtained by Le Bail fitting of powder X‐ray diffraction data;

^f)^ Solved from single crystal X‐ray diffraction data collected at 298 K after cooling a sample obtained by in situ desolvation of a pyridine solvate at high temperature;

^g)^ Solved from single crystal X‐ray diffraction data collected at 150 K;

^h)^ Refined from powder X‐ray or synchrotron diffraction data;

^i)^ Solved from single crystal X‐ray diffraction data collected at 150 K.

As well as providing highly useful structural information, it has not escaped our attention that the conditions within the electron diffractometer sample environment – cryogenic temperatures and microbar vacuum – mimic those prior to common gas adsorption experiments. Crystal structures obtained at 100 K, therefore, could be used as a proxy for the phase present prior to carrying out benchmark N_2_ adsorption–desorption isotherms at 77 K.^[^ ^58^ ^]^ Across our experiments, we were unable to isolate a particle of MIL‐53(Cr) in the anhydrous, fully closed pore phase, which may explain why MIL‐53(Cr) adsorbs significant quantities of N_2_ at 77 K whilst other MIL‐53(M) homologues typically do not.^[^ ^59^ ^]^ We therefore expect that, in future, variable temperature electron diffraction in specific environmental sample chambers could be used to elucidate structural changes during gas adsorption in the same way that in situ powder X‐ray diffraction^[^ ^60^ ^]^ has revolutionised our understanding of breathing and highly unusual phenomena like negative gas adsorption^[^ ^61^ ^]^ in synthetic porous materials.

## Conflict of Interest

The authors declare no conflict of interest.

## Supporting information



Supporting Information

## Data Availability

CCDC 2470910 – 2470924 contain the supplementary crystallographic data for this paper. These data can be obtained free of charge from The Cambridge Crystallographic Data Centre via https://www.ccdc.cam.ac.uk/structures/. The remainder of the data that support the findings of this study can be obtained free of charge at https://doi.org/10.5525/gla.researchdata.2080.
