# Supplementary material for: Observing Transient Breathing States of MIL‐53 Homologues Using In Situ Single Crystal 3D Electron Diffraction
Source: Small. 2025 Oct 30;21(50):e09071. doi: 10.1002/smll.202509071 (PMC12710115; doi:10.1002/smll.202509071)
Supplement: Supplementary file 1 — Supporting Information [file SMLL-21-e09071-s001.pdf]

NANO · MICRO small

Supporting Information

for *Small*, DOI 10.1002/smll.202509071

Observing Transient Breathing States of MIL-53 Homologues Using In Situ Single Crystal 3D Electron Diffraction

*Matthew Liddle, Celine Beck, Russell M. Main, Dominic Bara, Claire Wilson, Donald A. MacLaren, David Boldrin and Ross S. Forgan**

Observing Transient Breathing States of MIL-53 Homologues using *in situ* Single Crystal Electron Diffraction

Matthew Liddle,[†,1] Celine Beck,[†,2,3] Russell M. Main,[1] Dominic Bara,[1] Claire Wilson,[1] Donald A. MacLaren,[2] David Boldrin,[2] and Ross S. Forgan[*,1]

[†]These authors contributed equally

1. School of Chemistry, University of Glasgow, Glasgow G12 8QQ, UK.

Email: ross.forgan@glasgow.ac.uk

2. SUPA, School of Physics and Astronomy, University of Glasgow, Glasgow G12 8QQ, UK.

3. I11 High-resolution Powder Diffraction Beamline, Diamond Light Source Ltd, Harwell Science and Innovation Campus, Didcot, Oxfordshire, OX11 0DE, UK.

SUPPORTING INFORMATION

Table of Contents

S1. Material and Methods **S2.**

S2. Nomenclature **S4.**

S3. Synthesis **S5.**

S3.1. MIL-53(Cr) **S5.**

S3.2. MIL-53(Ga) **S5.**

S4. Variable Temperature Powder X-Ray Diffraction **S7.**

S4.1. MIL-53(Cr) **S7.**

S4.2. MIL-53(Ga) **S11.**

S5. Single Crystal Electron Diffraction **S16.**

S6. Crystallographic Data Tables **S24.**

S6.1. MIL-53(Cr) **S24.**

S6.2. MIL-53(Ga) **S38.**

S7. References **S54.**

S1. Material and Methods

Powder X-Ray Diffraction: variable temperature powder X-ray diffraction data for MIL-53(Cr) were collected on a PANalytical X'Pert PRO diffractometer (λ ($CuK\alpha_1$) = 1.5405 Å) using an Anton Paar HTK-1200N heating stage. For MIL-53(Ga), diffraction data were collected on a Malvern Panalytical Empyrean diffractometer (λ (CuKα) = 1.5418 Å). Synthetic data were collected at 298 K using reflection-transmission spinner 3.0 stage and variable temperature diffraction data were collected using an Anton Paar XRK-900 heating stage. Selected diffractograms were fitted using Jana 2020.[S1]

3D-ED Single Crystal Electron Diffraction: for structural characterization with 3D-ED, powder samples were deposited (for MIL-53(Cr) in a drop of ethanol and for MIL-53(Ga) as a powder shaken with the grid) on a 200-mesh copper TEM grid coated with a continuous or lacey film of ultrathin amorphous carbon. Many well-diffracting crystalline grains were found in each case; see Figure S1 for a snapshot of a representative grain of MIL-53(Cr). Short (~2 min) collection times combined with the low beam energy meant that beam damage to samples was not observed during data collection.

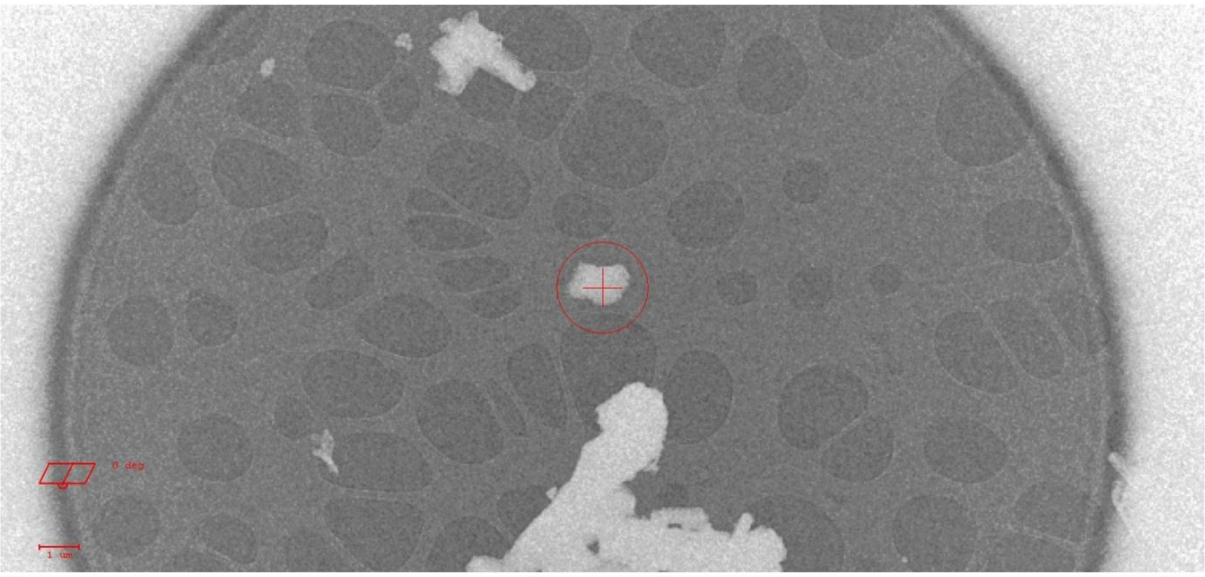

Figure S1. Image showing a crystal of MIL-53(Cr) used for 3D-ED data collection at 175K, the red circle shows the selected aperture area illuminated and the scale bar.

3D-ED measurements were performed on a Rigaku XtaLAB Synergy-ED[S2] equipped with a LaB_6 source operating at 200 kV (λ= 0.0251 Å) at 10^{-5} Pa, and at a range of temperatures, between 100 and 327 K, using a Gatan Elsa Cryoholder. Where data were collected at low temperature the samples were cryo-transferred to the cryoholder and into the diffractometer at 175 K. A series of diffraction patterns were collected for several crystals at each temperature using a Rigaku HyPix-ED detector during continuous rotation of the crystals over ca. 120 °, with each image having a rotation width of 0.25 ° and an exposure time of a few seconds per degree, using a selected area aperture with an apparent diameter of 2 µm. The program CrysAlisPro[S3] was used to control the data collection and to process the diffraction data, for indexing, data reduction and space group determination.

Crystallographic details are contained in the deposited CIFs (CCDC 2470910–2470924) for each of the structures reported and selected details are given below. The crystal structures were solved *ab initio* with SHELXT[S4] and least-squares refinement in kinematic approximation was performed with SHELXL[S5] or olex2.refine[S6] using scattering factors for electrons.[S7] An extinction parameter was refined to mitigate the effects of multiple diffraction. Anisotropic atomic displacement parameters (ADPs) were refined for non-hydrogen atoms except for the lattice water oxygen atoms, while H atoms were a mix of being placed in geometrically idealized position and refined as part of a riding model, using tabulated distances from neutron diffraction[S8] and isotropic thermal parameters derived from the parent atom, or in some cases located in difference Fourier maps and U_{iso} values refined with O-H distances restrained to distances from neutron diffraction.

CCDC 2470910–2470924 contain the supplementary crystallographic data for this paper. These data can be obtained free of charge from The Cambridge Crystallographic Data Centre via www.ccdc.cam.ac.uk/structures.

S2. Nomenclature

The nomenclature used to describe the various states of MIL-53(M) across the different homologues is inconsistent, which leads to confusion when trying to compare similar structures with differing metals. To remedy this, we have used a universal nomenclature scheme in this study, which is laid out in Table S1.

Table S1. The naming scheme applied in this study in comparison to examples of the varying conventions for naming MIL-53 phases in the literature.

**Universal Nomenclature**	**Structure Description**	**Example Literature Nomenclature**
**MIL-53(M)_cp_mh**	The closed pore (cp) monohydrate (mh) structure with overall composition [M(OH)(BDC)]·H_2O. Typically formed by activating samples and exposing them to ambient moisture. Examples given in Table S2.	MIL-53(Cr)_lt; MIL-53(Al) *lt;* MIL-53(Al)LT;
**MIL-53(M)_cp_ah**	The closed pore (cp) anhydrous (ah) structure, typically isolated by activating samples at different temperatures. Examples are given in Table S3.	MIL-53(Fe)_ht; MIL-53(Ga)_lt
**MIL-53(M)_op_ah**	The open pore (op) anhydrous structure, typically found by heating samples above the boiling point of bound solvent. Not known for all homologues (e.g., M = Fe). Examples are given in Table S4.	MIL-53(Cr)_ht; MIL-53(Ga)_ht; MIL-53(Al)HT

S2. Synthesis

S2.1. MIL-53(Cr)

MIL-53(Cr) was synthesised according to our previously published hydrothermal methodology using HCl as modulator.[S9] The sample was isolated as the closed pore monohydrate MIL-53(Cr)_cp_mh (previously described as MIL-53(Cr)_lt) by sequential activation in *N*,*N*-dimethylformamide (DMF) and MeOH, followed by heating under high vacuum. The sample was ground lightly in a mortar and pestle prior to analysis by 3D-ED to de-aggregate individual grains.

S2.2. MIL-53(Ga)

$Ga(NO_3)_3{\cdot}6H_2O$ (138.7 mg, 0.5 mmol) and terephthalic acid (166.1 mg, 1 mmol) were placed in a Pyrex glass jar (50 mL) to which DMF (10 mL) was added. The mixture was sonicated for 5 min until a homogeneous solution was observed. The solution was then transferred to a Teflon-lined acid digestion vessel (Parr, 45 mL) and placed in an isothermal oven at 220 °C for 6 h. After cooling to room temperature, the solids were collected by centrifugation (4500 rpm for 15 min) and washed with DMF (3 ×15 mL) using centrifuge-wash cycles. The product was then dried under vacuum and characterised by powder-X-ray diffraction (PXRD, Figure S2).

The sample was activated by heating to 50 °C (ramp rate = 5 °C min^{-1}; held at 50 °C for 30 min), then further heated to 220 °C (ramp rate = 10 °C min^{-1}) and held for 24 h at 220 °C under reduced pressure (evacuation rate = 6.7 mbar s^{-1}, pressure = 2 $\times 10^{-5}$ bar). The activated sample was exposed to air and analysed by PXRD (Figure S2), confirming the isolation of MIL-53(Ga) as the closed pore monohydrate phase, MIL-53(Ga)_cp_mh, which is isostructural to MIL-53(Cr)_cp_mh.

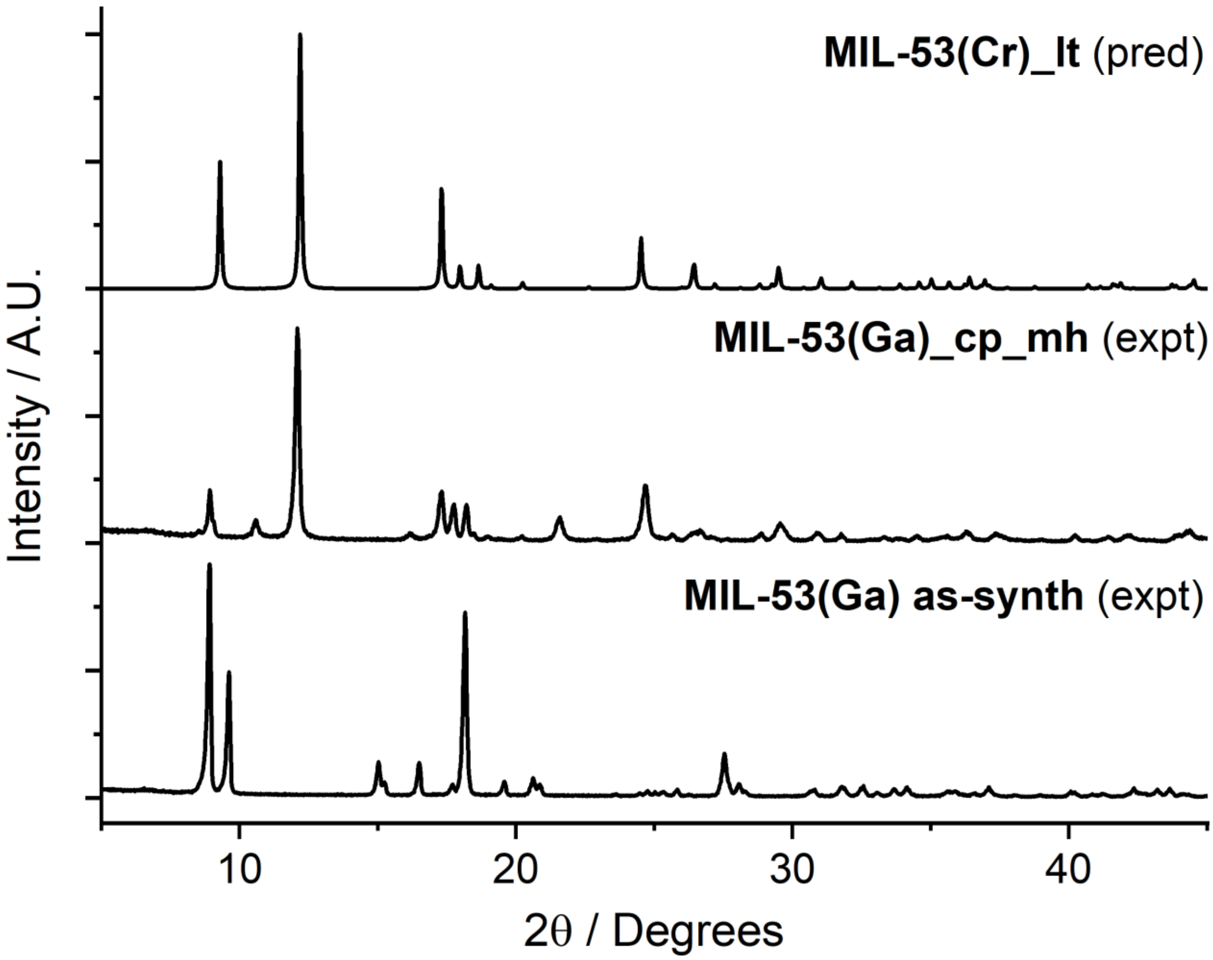

Figure S2. Stacked powder X-ray diffractograms of MIL-53(Ga) as synthesised (DMF solvate) and MIL-53(Ga)_cp_mh compared to the pattern predicted for MIL-53(Cr)_cp_mh (the closed pore monohydrate form known in the literature as MIL-53(Cr)_lt).[S10]

S3. Variable Temperature Powder X-Ray Diffraction

Selected diffractograms from the variable temperature PXRD analyses of MIL-53(Cr) and MIL-53(Ga) underwent Le Bail fitting using Jana 2020[S1] – the peak shape function chosen was Pseudo-Voigt and zero shift correction were performed – to determine unit cell parameters of:

- The closed pore monohydrated phases (previously referred to as MIL-53(Cr)_lt). For simplicity here, we denote these with the suffix cp_mh.
- The closed pore anhydrous phases (not previously observed for MIL-53(Cr) but referred to as MIL-53(Ga)_lt). For simplicity here, we denote these with the suffix cp_ah.
- The open pore anhydrous phases (previously referred to as MIL-53(Cr)_ht and MIL-53(Ga)_ht). For simplicity here, we denote these with the suffix op_ah.

S3.1. MIL-53(Cr)

The full variable temperature powder X-ray diffraction dataset is provided in Figure S3. Le Bail fitting of selected data sets was used to confirm the formation of MIL-53(Cr)_cp_mh (Figure S4), MIL-53(Cr)_cp_ah (Figure S5) and MIL-53(Cr)_op_ah (Figure S6).

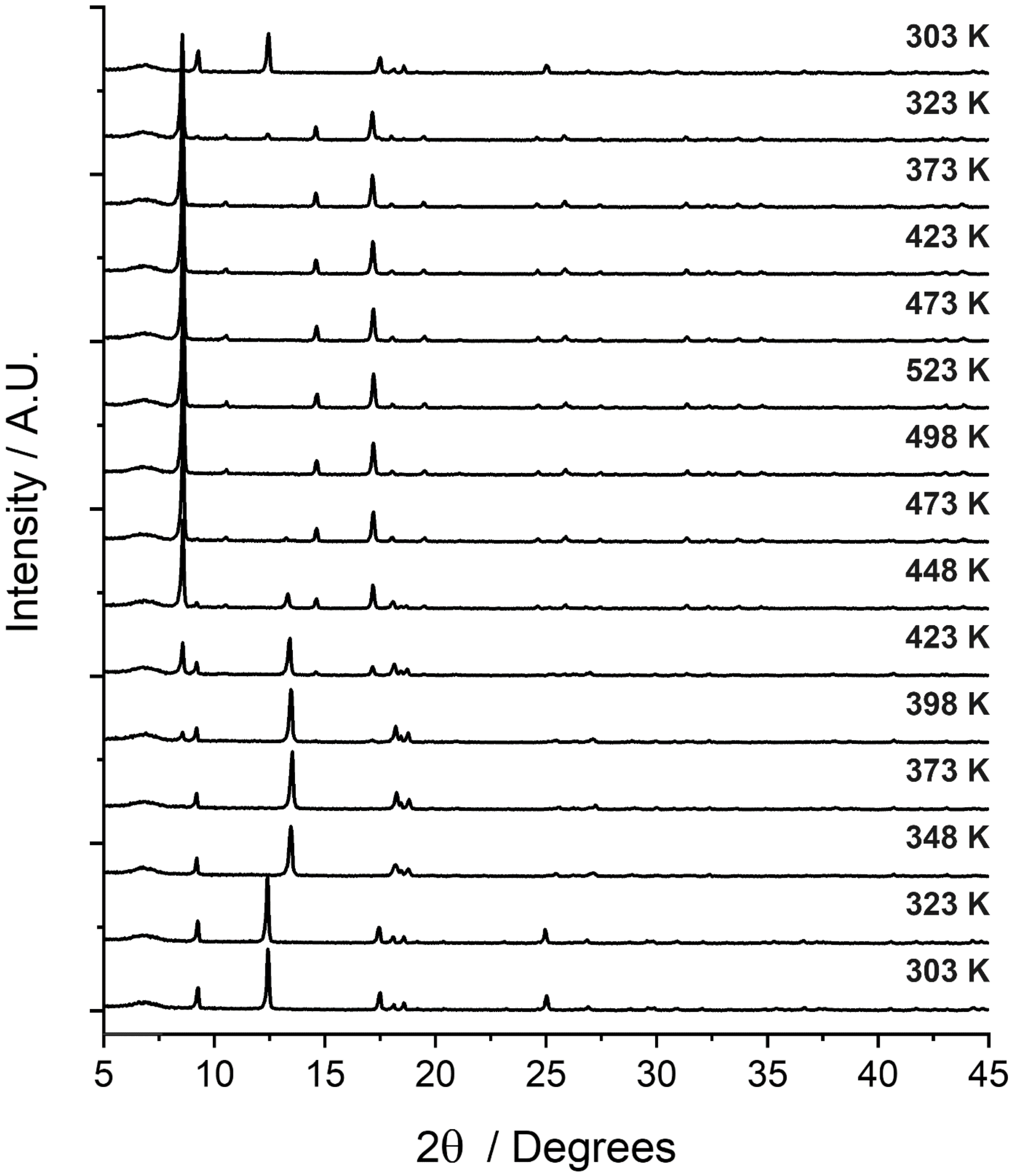

Figure S3. Stacked variable temperature powder X-ray diffractograms of MIL-53(Cr)_cp_mh heating (bottom to top) from 303 – 523 K then cooling back to 303 K. The broad signal at 2θ ~ 7 ° comes from the sample holder.

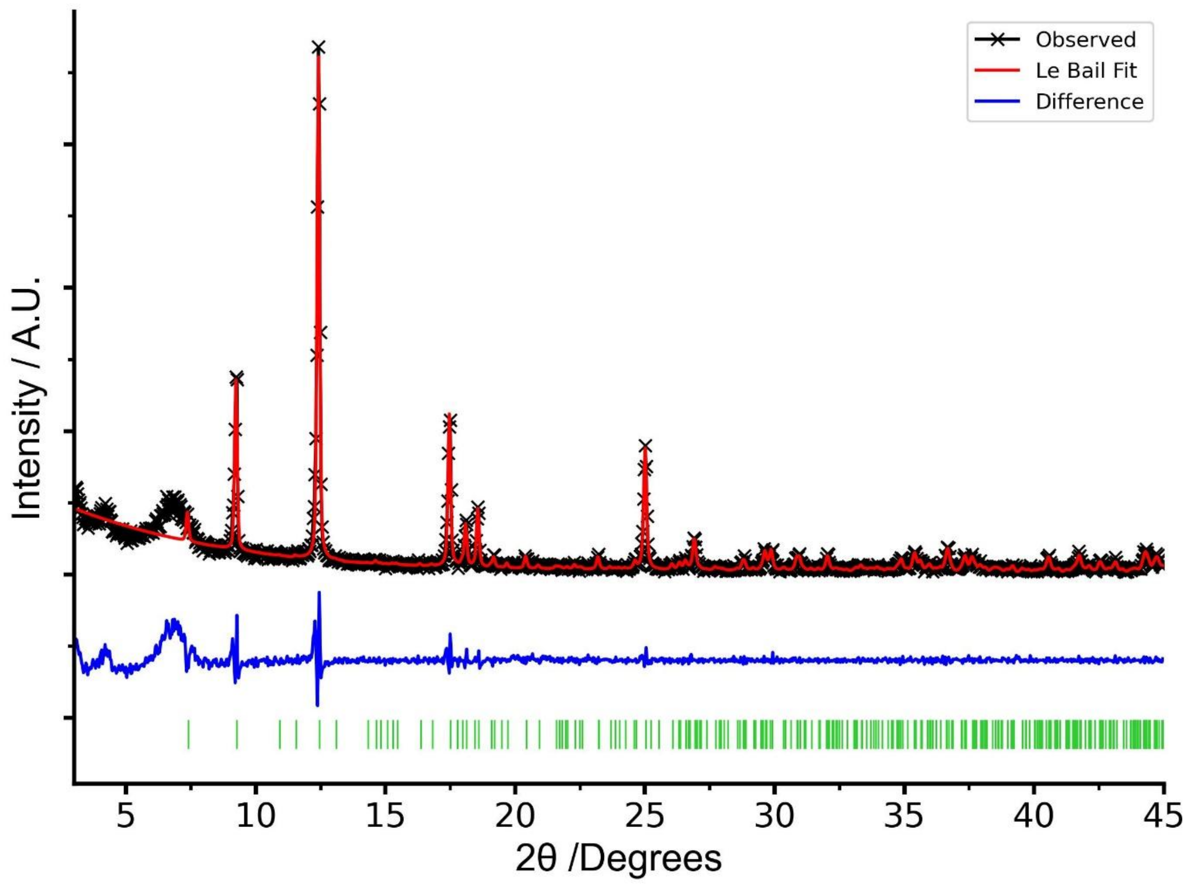

Figure S4. Le Bail fit of a powder X-ray diffractogram of MIL-53(Cr)_cp_mh (known in the literature as MIL-53(Cr)_lt) collected at 303 K. Le Bail fit: $P2_1/c$, a = 6.78769 Å, b = 15.31669 Å, c = 19.16767 Å, β = 95.67698 °, V = 1982.991 Å^3, R_p = 16.10, wR_p = 20.87.

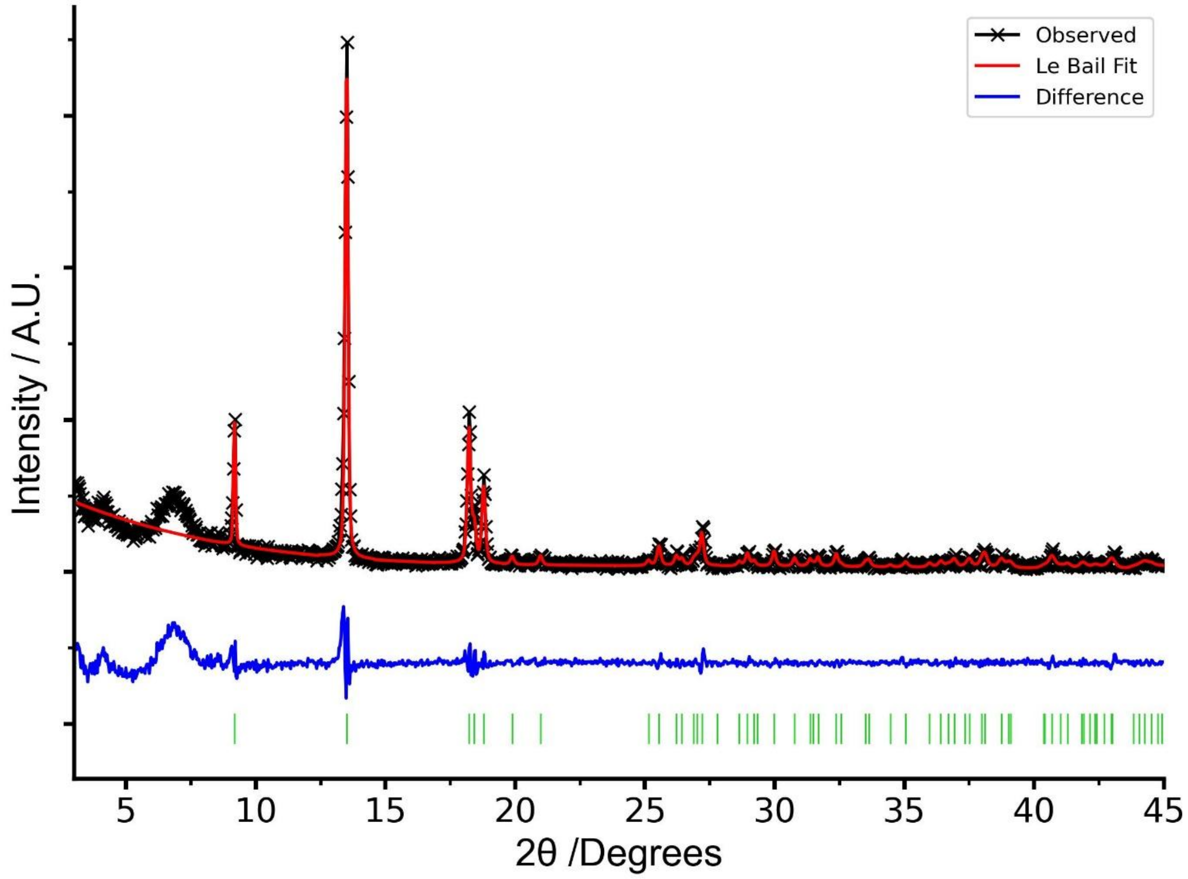

Figure S5. Le Bail fit of a powder X-ray diffractogram of MIL-53(Cr)_cp_ah collected at 373 K. Le Bail fit: *I*2/*a*, *a* = 6.823285 Å, *b* = 6.969891 Å, *c* = 19.33180 Å, *β* = 95.25928 °, *V* = 915.5026 $Å^3$, R_p = 17.62, wR_p = 22.48.

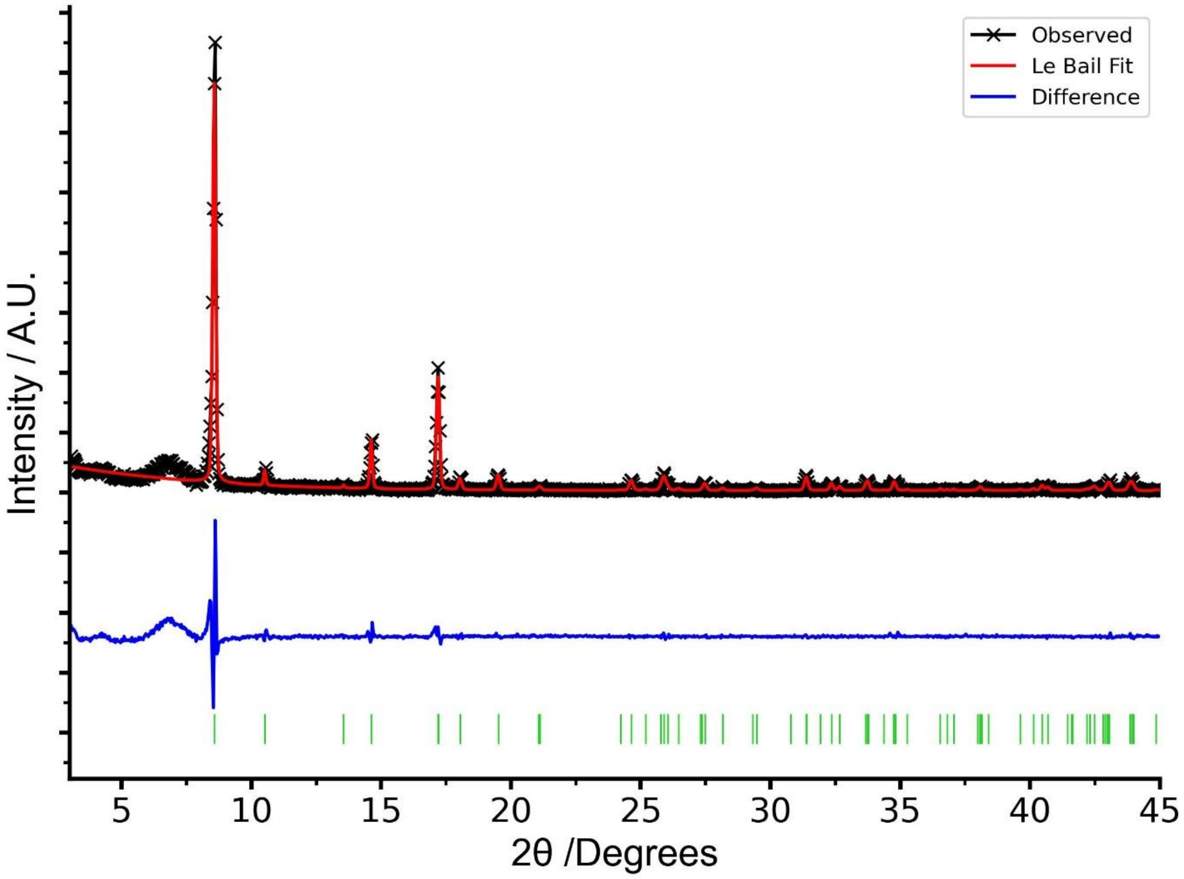

Figure S6. Le Bail fit of a powder X-ray diffractogram of MIL-53(Cr)_op_ah (known in the literature as MIL-53(Cr)_ht) collected at 523 K. Le Bail fit: *Imma*, a = 16.81174 Å, b = 6.836602 Å, c = 13.05933 Å, V = 1500.975 $Å^3$, R_p = 19.62, wR_p = 25.11.

S3.2. MIL-53(Ga)

The full variable temperature powder X-ray diffraction dataset is provided in Figure S7. Le Bail fitting of selected data sets was used to confirm the formation of MIL-53(Ga)_cp_mh (Figure S8), MIL-53(Ga)_cp_ah (Figure S9) and MIL-53(Ga)_op_ah (Figure S10). Minor Bragg peaks at 2θ ~ 10.5 ° and 16 ° were observed across all diffractograms during the variable temperature powder diffraction experiments (Figure S7) and were not fitted, as we expect they correspond to a minor phase impurity that is only noticeable when the sample diffracts weakly.

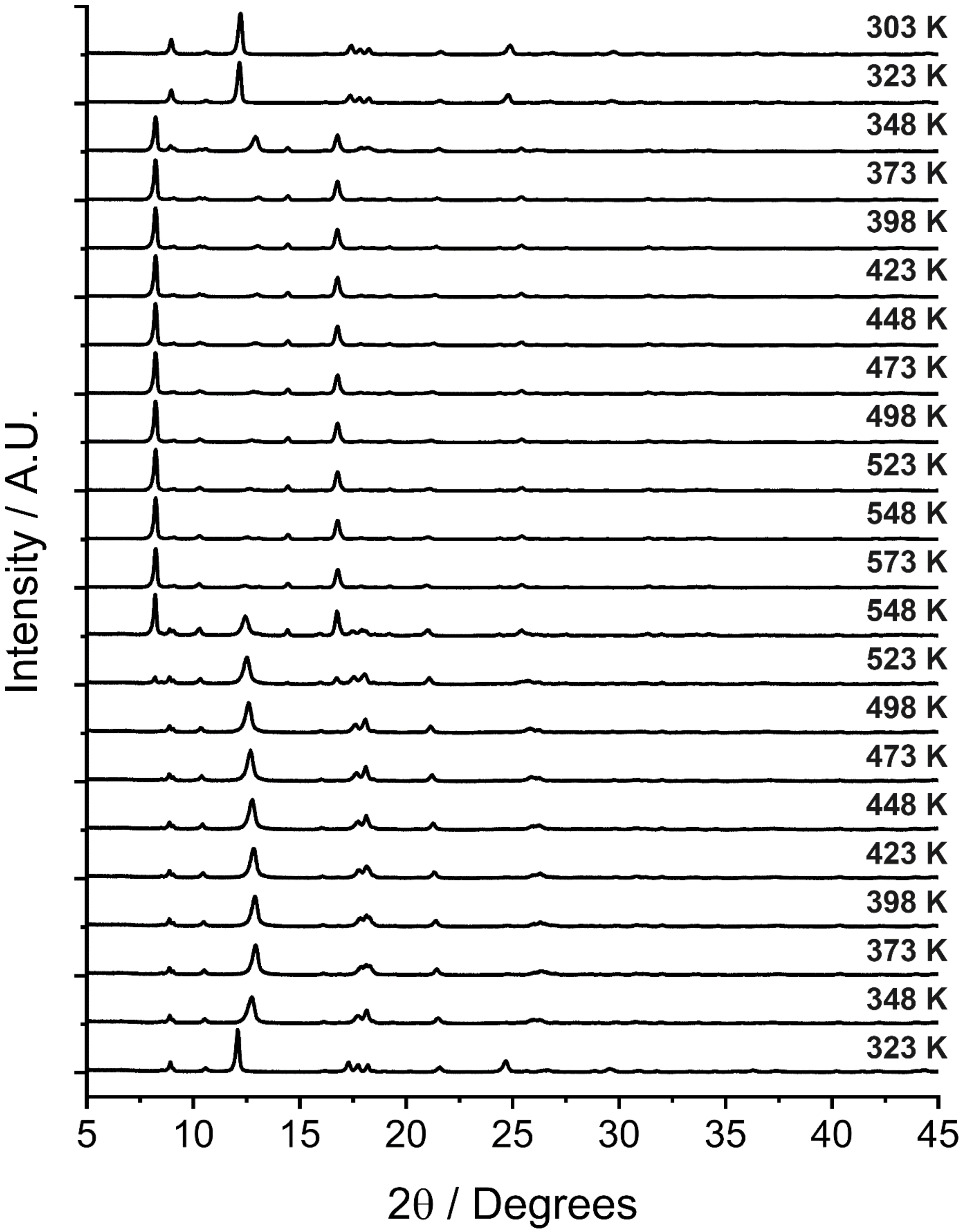

Figure S7. Stacked variable temperature powder X-ray diffractograms of MIL-53(Ga)_cp_mh heating (bottom to top) from 323 – 573 K then cooling back to 303 K.

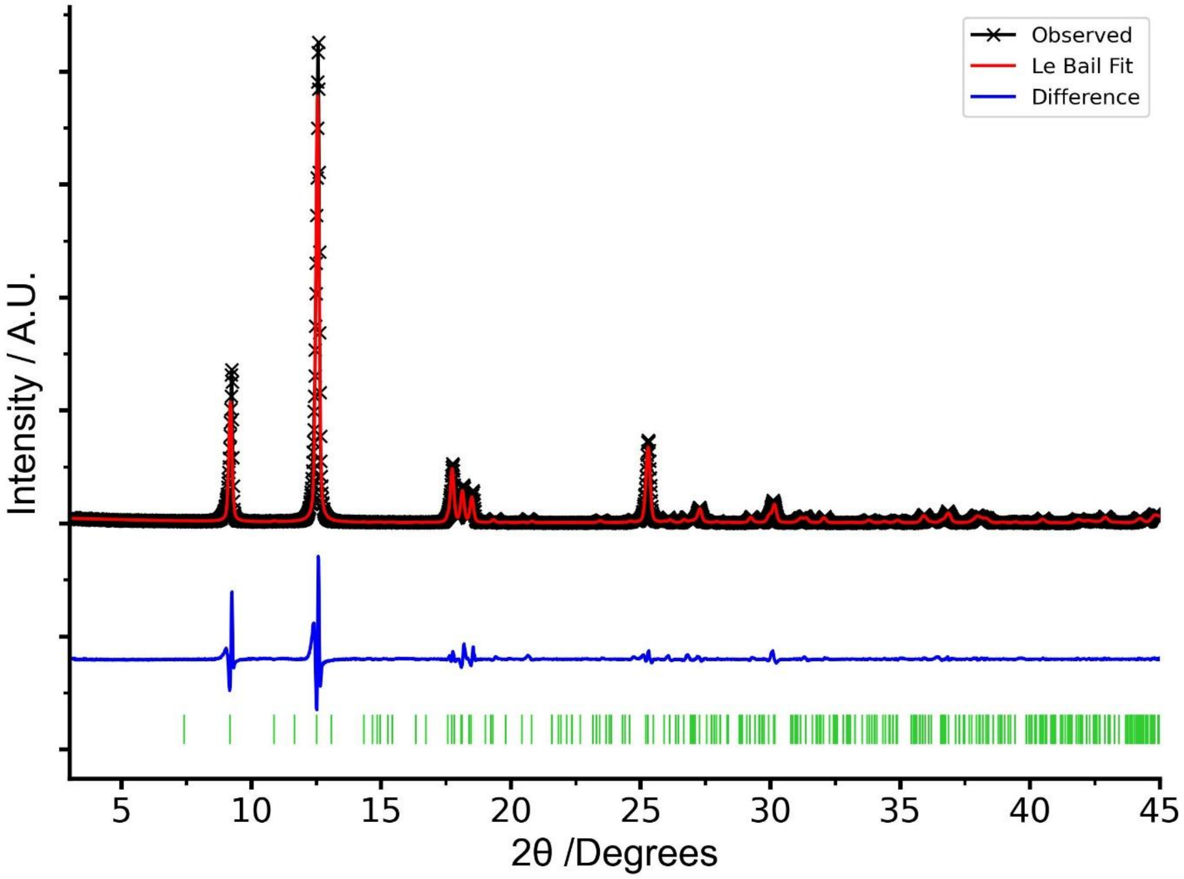

Figure S8. Le Bail fit of a powder X-ray diffractogram of MIL-53(Ga)_cp_mh collected at 323 K. Le Bail fit: $P2_1/c$, a = 6.793119 Å, b = 15.18997 Å, c = 19.38056 Å, β = 95.54301 °, V = 1990.476 Å^3, R_p = 18.07, wR_p = 23.47.

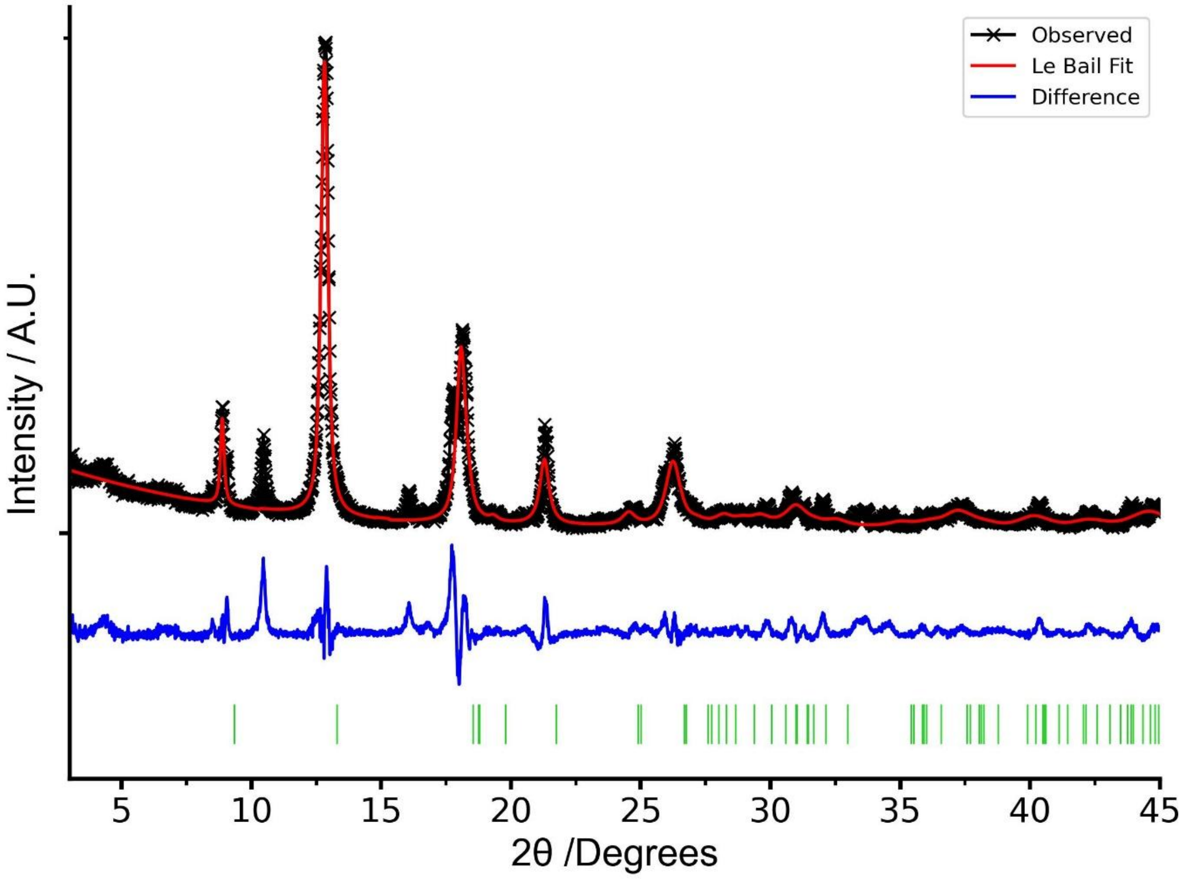

Figure S9. Le Bail fit of a powder X-ray diffractogram of MIL-53(Ga)_cp_ah (known in the literature as MIL-53(Ga)_lt) collected at 398 K. Le Bail fit: *I*2/*a*, *a* = 6.543624 Å, *b* = 7.414697 Å, *c* = 20.12531 Å, *β* = 94.33310 °, *V* = 973.6688 Å^3, R_p = 18.20, wR_p = 24.39. Note the Bragg peaks at 2θ ~ 10.5 ° and 16 ° were observed across all diffractograms during the variable temperature powder diffraction experiments (see Figure S7) and hence were not included in the final refinement of MIL-53(Ga)_cp_ah. It is more prominent in this diffractogram due to the lower intensity observed for this particular run.

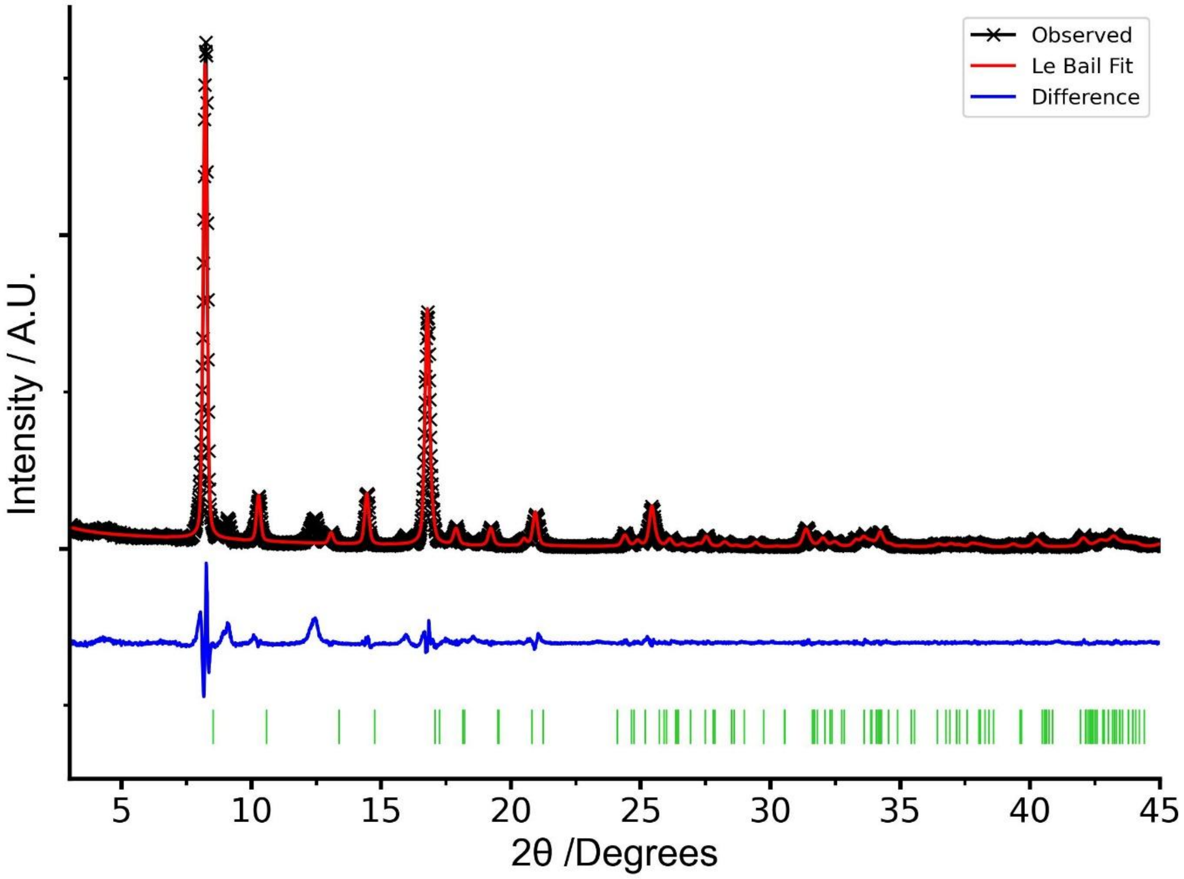

Figure S10. Le Bail fit of a powder X-ray diffractogram of MIL-53(Ga)_op_ah (known in the literature as MIL-53(Ga)_ht) collected at 573 K. Le Bail fit: *I*2/*a*, *a* = 6.737216 Å, *b* = 13.24043 Å, *c* = 16.72498 Å, *β* = 89.72912 °, *V* = 1491.92 Å^3, R_p = 13.93, wR_p = 20.40.

S4. Single Crystal Electron Diffraction (3D-ED)

The crystallographic data obtained by electron diffraction for the closed pore monohydrated MIL-53(Cr) and MIL-53(Ga) samples are compared with literature data in Table S2.

Table S2. Collation of unit cell data for structures of closed pore, hydrated MIL-53(M)_cp_mh materials. Structures are refined from X-ray (laboratory or synchrotron) powder diffraction data collected at room temperature unless stated. Our single crystal structures from electron diffraction (3D-ED) data are listed in bold on the bottom rows.

M	SG[a]	*a* / Å	*b* / Å	*c* / Å	*β* / °	*V* / Å^3	V_c / Å^3[b]	ref
Cr	*C*2/*c* (15)	19.685(4)	7.849(1)	6.782(1)	104.90(1)	1012.64	1012.6	S10]
Cr	*C*2/*c* (15)	20.9168(2)	7.7005(1)	6.7780(2)	114.362(1)	994.521	994.5	[S11]
Fe	*P*2$_1$/*c* (14)	19.3197	15.0362	6.8351	96.305	1973.5	986.8	[S12]
Fe	*C*2/*c* (14)	21.12992(9)	7.64271(6)	6.83058(7)	114.9352(4)	1000.25(2)	1000.3	[S13]
Al	*Cc* (9)	19.513(2)	7.612(1)	6.576(1)	104.24(1)	946.74(1)	946.7	[S14]
Al[c]	*Cc* (9)	19.55(2)	7.81(2)	6.62(2)	104.5(2)	978.582	978.5	[S15]
Al	*P*2$_1$/*c* (14)	19.5042(3)	15.2014(1)	6.5693(1)	104.1797(13)	1888.40(4)	944.2	[S16]
Al[c]	*P*2$_1$/*n* (14)	19.548	15.013	6.655	103.69	1897.58	948.8	[S15]
Ga	*P*2$_1$/*c* (14)	19.6801(2)	15.1165(1)	6.6716(1)	103.7573(9)	1927.83(3)	963.9	[S16]
Ga	*Cc* (9)	19.6597(2)	7.6444(1)	6.6716(1)	103.8831(8)	973.36(2)	973.4	[S17]
Ga	*P*2$_1$/*c* (14)	19.7053(2)	15.1642(4)	6.68117(9)	103.7936(8)	1938.56(7)	969.3	[S18]
Ga[d]	*P*2$_1$/*c* (14)	6.680(1)	14.8550(7)	19.269(2)	96.224(3)	1900.8(3)	950.5	[S19]
**Cr[e]**	***P*2$_1$/*c* (14)**	**6.8355(10)**	**15.103(3)**	**19.3338(14)**	**96.039(11)**	**1985.2(5)**	**992.6**	**This work**
**Cr[e]**	***I*2/a (15)**	**6.7892(16)**	**7.517(5)**	**19.202(4)**	**95.80(2)**	**975.0(7)**	**975.0**	**This work**
**Ga[e]**	***P*2$_1$/*c* (14)**	**6.7487(3)**	**15.1816(18)**	**19.5011(10)**	**95.922(5)**	**1987.3(3)**	**993.7**	**This work**
**Ga[e]**	***I*2/a (15)**	**6.6980(18)**	**7.498(6)**	**19.239(5)**	**96.20(3)**	**960.6(9)**	**960.6**	**This work**

[a]SG = Space Group number and setting.

[b]*P*-centred unit cell volumes halved to allow comparison with *C*- and *I*-centred unit cells.

[c]Solved from electron diffraction data collected at 293 K.

[d]Solved from single crystal X-ray diffraction data collected at 150 K.

[e]Solved from single crystal electron diffraction data collected at 175 K.

Packing images of the primitive and *I*-centred monoclinic structures of the closed pore monohydrates of MIL-53(Cr) and MIL-53(Ga) solved by electron diffraction are presented for comparison in Figure S11.

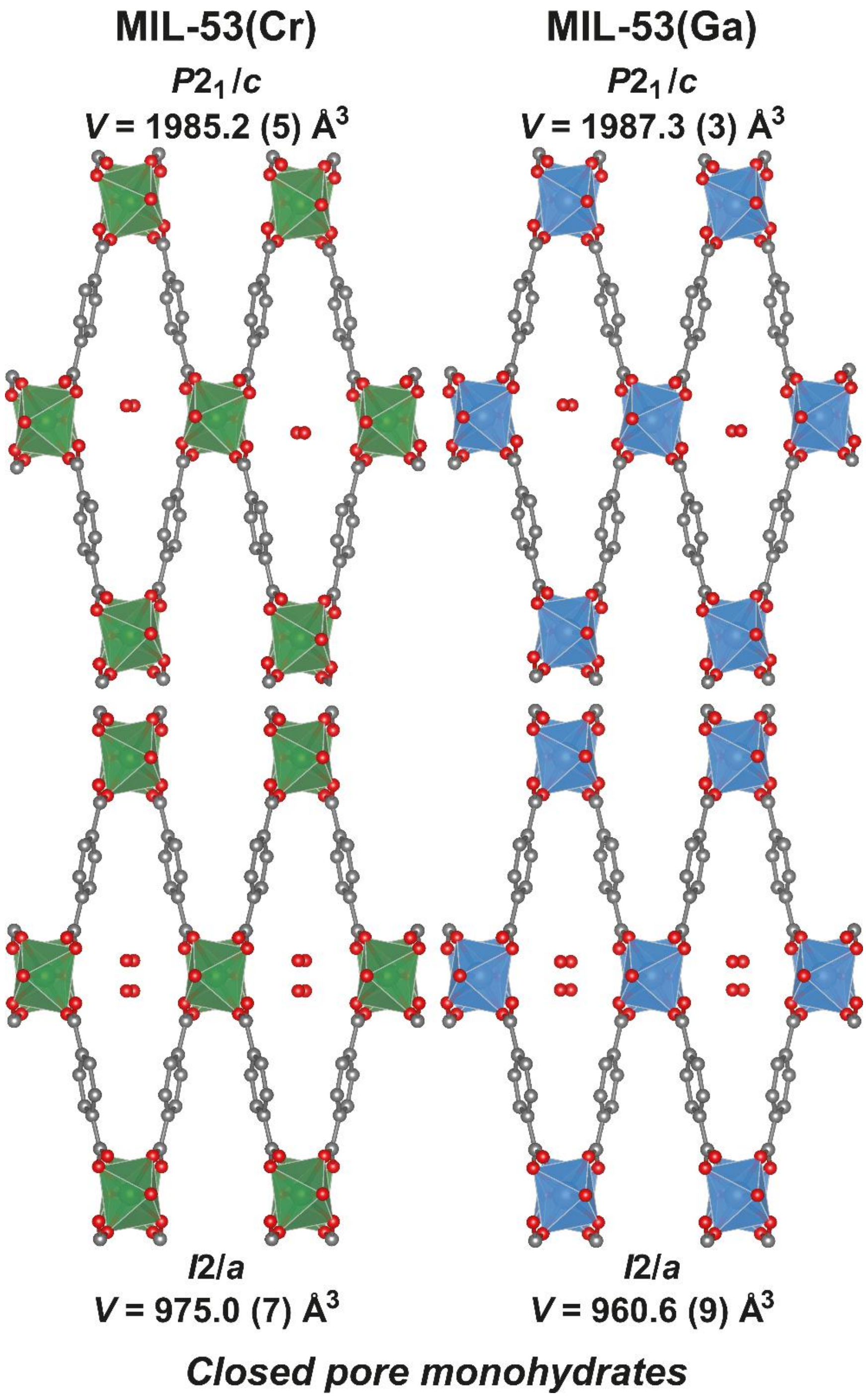

Figure S11. Comparison of the crystal structures of the primitive and *I*-centred monoclinic structures of the closed pore monohydrates of MIL-53(Cr) and MIL-53(Ga) solved using electron diffraction data, showing their close similarities.

The crystallographic data obtained by electron diffraction for the open pore anhydrous MIL-53(Cr)_op_ah sample are compared with literature data in Table S3.

Table S3. Collation of unit cell data for structures of open pore, anhydrous MIL-53(M)_op_ah materials. Structures are refined from synchrotron or X-ray powder diffraction data collected at room temperature unless stated. Our single crystal structure from electron diffraction is listed in the bottom row.

M	SG[a]	*a* / Å	*b* / Å	*c* / Å	*V* / Å³	ref
Cr[b]	*Imcm* (74)	6.812(1)	16.733(1)	13.038(1)	1486.14	[S10]
Al	*Imma* (74)	6.6085(9)	16.675(3)	12.813(2)	1411.95	[S14]
Al	*Imma* (74)	6.6361(5)	16.744(2)	12.847(2)	1427.5(3)	[S20]
Al[c]	*Imma* (74)	6.62	16.73	12.89	1427.6	[S15]
Al[c]	*Imma* (74)	6.65	17.27	12.54	1440.16	[S15]
Al[c]	*Imma* (74)	6.61(3)	17.25(3)	12.81(1)	1460.63	[S15]
Ga	*Imma* (74)	6.7166	16.6784	13.2093	1479.7	[S18]
Ga[b][d]	*Imma* (74)	6.7401(7)	16.7753(17)	13.2673(18)	1500.1(3)	[S19]
**Cr[e]**	***Imma* (74)**	**6.8369(12)**	**16.674(6)**	**13.159(9)**	**1500.1(12)**	**This work**

[a]SG = Space Group and setting (all space group number 74).

[b]*a*, *b* and *c* reordered compared to published order to allow comparison between unit cells published order (Cr *bca*, Ga *bac*).

[c]Solved from electron diffraction data collected at 293 K.

[d]Solved from single crystal X-ray diffraction data collected at 473 K by *in situ* desolvation of a pyridine solvate.

[e]Solved from single crystal electron diffraction data collected at 298 K.

The crystallographic data obtained by electron diffraction for the closed pore anhydrous MIL-53(Ga)_cp_ah sample are compared with literature data in Table S4.

Table S4. Collation of unit cell data for structures of closed pore, anhydrous MIL-53(M)_cp_ah materials. Structures are refined from synchrotron or X-ray powder diffraction data collected at room temperature unless stated. Our single crystal structure from electron diffraction is listed in the bottom row.

M	SG[a]	*a* / Å	*b* / Å	*c* / Å	*β* / °	*V* / Å³	ref
Fe	*C*2/*c* (15)	21.2693	6.7589	6.8838	114.625	899.6	[S12]
Ga	*C*2/*c* (15)	19.8331(2)	6.8556(4)	6.7143(2)	103.8752(9)	886.28(5)	[S18]
Ga[b]	*Cc* (9)	19.7749(2)	6.96751(8)	6.70007(8)	103.943(1)	895.95(2)	[S17]
Ga[c]	*I*2/*a* (15)	6.7061(8)	6.914(2)	19.322(2)	95.68(1)	891.5(3)	[S19]
Sc[d]	*C*2/*c* (15)	21.5050(3)	6.63008(7)	7.2743(2)	113.543(1)	950.838(3)	[S21]
**Ga[e]**	***I*2/*a* (15)**	**6.7449(19)**	**6.959(8)**	**19.343(6)**	**95.57(3)**	**903.6(11)**	**This work**

[a]SG = Space Group setting and number.

[b]Unit cell parameters obtained by Le Bail fitting.

[c]Solved from single crystal X-ray diffraction data collected at 298 K after cooling a sample obtained by *in situ* desolvation of a pyridine solvate at high temperature.

[d]Powder diffraction data collected at 650 K.

[e]Solved from single crystal electron diffraction data collected at 298 K.

The unit cell data for all intermediate structures of MIL-53(Cr) and MIL-53(Ga) collected by electron diffraction are collated in Table S5.

Table S5. Collation of unit cell data for structures of intermediate structures of MIL-53(M) collected by electron diffraction in this study.

M	SG[a]	*a* / Å	*b* / Å	*c* / Å	*β* / °	*V* / Å³	T / K
Ga	*I*2/*a* (15)	6.7570(12)	7.145(6)	19.569(3)	95.266(13)	940.8(8)	327
Ga	*I*2/*a* (15)	6.6980(6)	8.8946(13)	18.9895(14)	93.837(6)	1128.8(2)	175
Ga	*I*2/*a* (15)	6.7860(7)	8.962(3)	19.167(2)	94.111(10)	1162.7(4)	100
Ga	*I*2/*a* (15)	6.7672(7)	9.179(2)	19.1391(18)	93.136(8)	1187.1(3)	327
Ga	*I*2/*a* (15)	6.632(5)	10.12(2)	18.07(6)	92.31(11)	1212(5)	100
Cr	*Imma* (74)	18.449(19)	6.821(3)	10.29(3)	90	1295(5)	240
Cr	*Imma* (74)	6.8514(12)	18.143(9)	10.719(9)	90	1322.4(13)	300
Cr	*Imma* (74)	18.254(14)	6.890(4)	10.97(3)	90	1379(4)	100
Cr	*Imma* (74)	18.347(11)	6.941(2)	11.088(17)	90	1412(2)	300

[a]SG = Space Group setting and number.

The normalised unit cell data for all structures of MIL-53(Cr) and MIL-53(Ga) collected by electron diffraction are collated in Table S6. This allows comparison across samples which solved in differing space groups or with axes swapped. In addition, the pore angle Ψ, which corresponds to the obtuse vertex of the rhombic pore, is provided as a measurement of pore opening (Ψ = 90 ° corresponds to a completely open pore, Figure 5a in the main manuscript).

Table S6. Normalised unit cell data for all structures of MIL-53(M) from electron diffraction data in this study.

M	SG[a]	*a'* / Å[b]	*b'* / Å[b][c]	*c'* / Å[b]	*β* / °	*V* / Å^3	V_c / Å^3[d]	Ψ / °[e]
Ga	*I*2/*a* (15)	6.7449(19)	6.959(8)	19.343(6)	95.57(3)	903.6(11)	903.6(11)	140.43
Ga	*I*2/*a* (15)	6.7570(12)	7.145(6)	19.569(3)	95.266(13)	940.8(8)	940.8(8)	139.88
Ga	*I*2/*a* (15)	6.6980(18)	7.498(6)	19.239(5)	96.20(3)	960.6(9)	960.6(9)	137.42
Cr	*I*2/*a* (15)	6.7892(16)	7.517(5)	19.202(4)	95.80(2)	975.0(7)	975.0(7)	137.24
Cr	$P2_1/c$ (14)	6.8355(10)	7.5515	19.3338(14)	96.039(11)	1985.2(5)	992.6	137.40
Ga	$P2_1/c$ (14)	6.7487(3)	7.5908	19.5011(10)	95.922(5)	1987.3(3)	993.7	137.56
Ga	*I*2/*a* (15)	6.6980(6)	8.8946(13)	18.9895(14)	93.837(6)	1128.8(2)	1128.8(2)	129.80
Ga	*I*2/*a* (15)	6.7860(7)	8.962(3)	19.167(2)	94.111(10)	1162.7(4)	1162.7(4)	129.88
Ga	*I*2/*a* (15)	6.7672(7)	9.179(2)	19.1391(18)	93.136(8)	1187.1(3)	1187.1(3)	128.76
Ga	*I*2/*a* (15)	6.632(5)	10.12(2)	18.07(6)	92.31(11)	1212(5)	1212(5)	121.50
Cr	*Imma* (74)	6.821(3)	10.29(3)	18.449(19)	90	1295(5)	1295(5)	121.70
Cr	*Imma* (74)	6.8514(12)	10.719(9)	18.143(9)	90	1322.4(13)	1322.4(13)	118.85
Cr	*Imma* (74)	6.890(4)	10.97(3)	18.254(14)	90	1379(4)	1379(4)	117.99
Cr	*Imma* (74)	6.941(2)	11.088(17)	18.347(11)	90	1412(2)	1412(2)	117.71
Cr	*Imma* (74)	6.8369(12)	13.159(9)	16.674(6)	90	1500.1(12)	1500.1(12)	103.44

[a]SG = Space Group setting and number.

[b]Some *Imma* space groups have had *a*, *b* and *c* reordered to allow comparison between unit cells.

[c]*P*-centred *b* values have been halved to allow comparison with *C*- and *I*-centred unit cells.

[d]*P*-centred unit cell volumes halved to allow comparison with *C*- and *I*-centred unit cells.

[e]The obtuse vertex of the rhombic pore, see Figure 5a of the manuscript.

Linear fits were applied to plots of Ψ vs *b′* (Figure S12), V_c vs *b′* (Figure S13), and Ψ vs V_c (Figure S14) to quantify the correlations between these parameters across all structures. These plots are the same data as in Figures 5b-5d in the main manuscript, where data are presented without the linear fits.

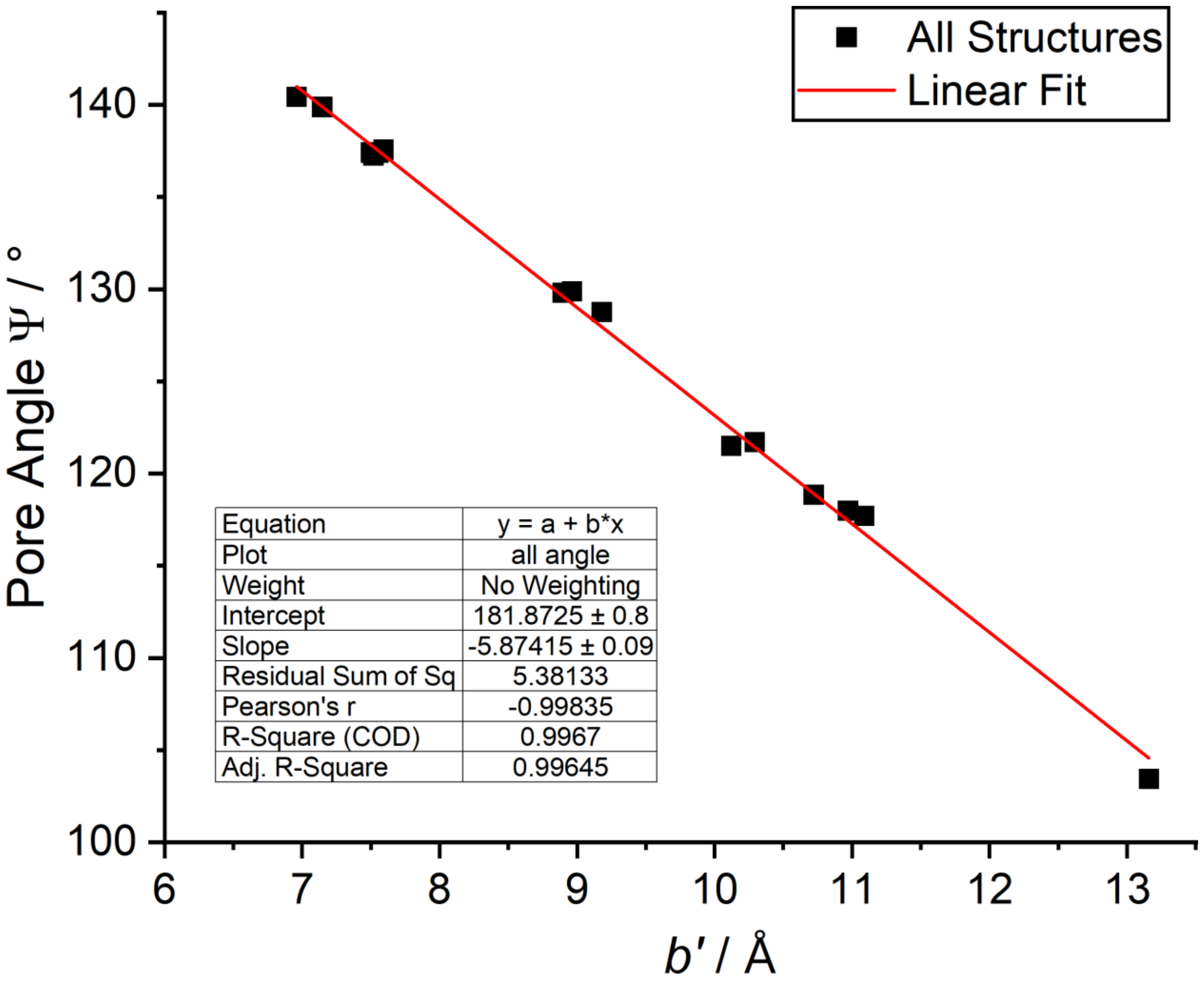

Figure S12. Plot of pore angle Ψ against normalised unit cell parameter *b′* for all structures of MIL-53(Cr) and MIL-53(Ga) collected by electron diffraction, with a linear data fit.

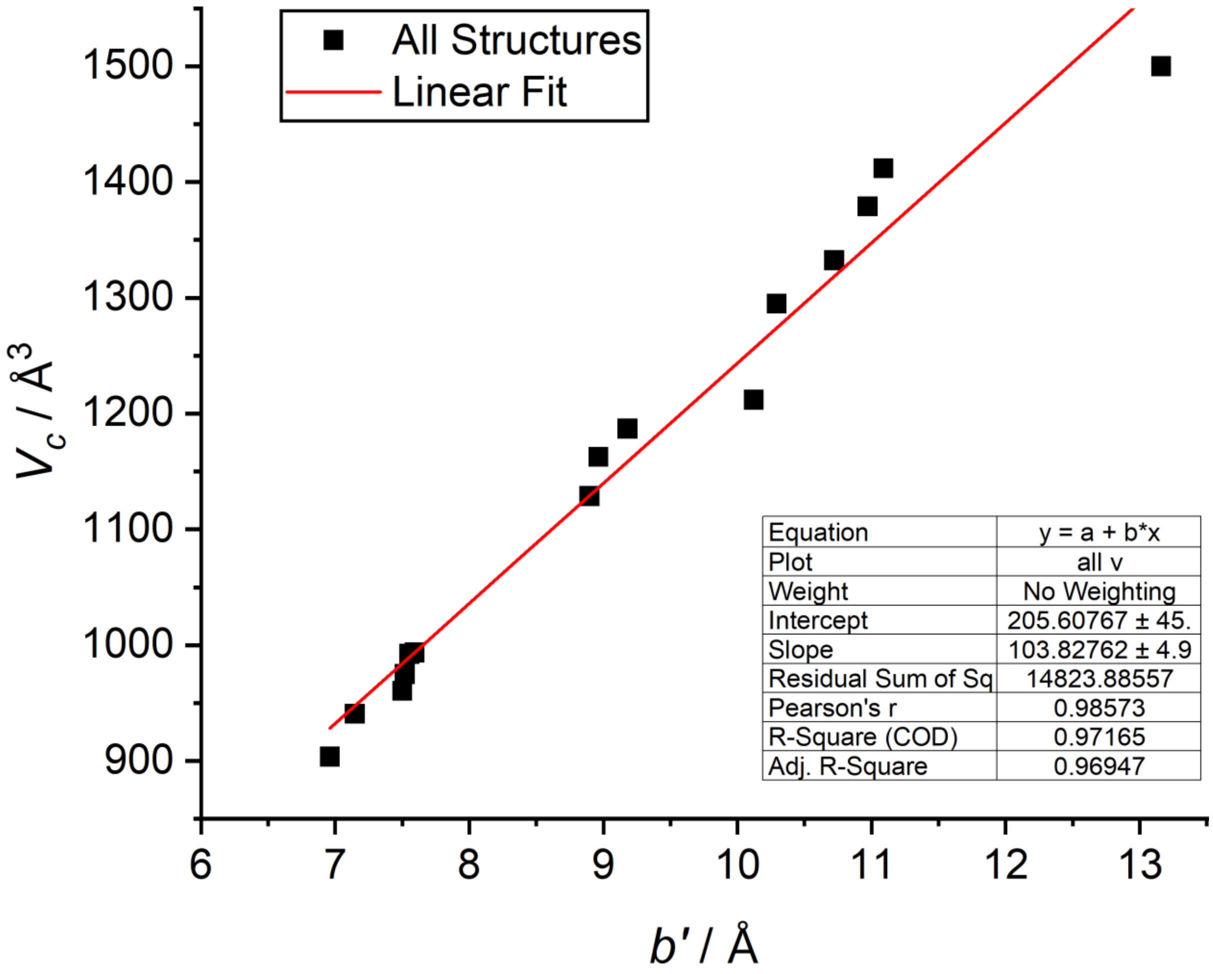

Figure S13. Plot of corrected unit cell volume V_c against normalised unit cell parameter *b'* for all structures of MIL-53(Cr) and MIL-53(Ga) collected by electron diffraction, with a linear data fit.

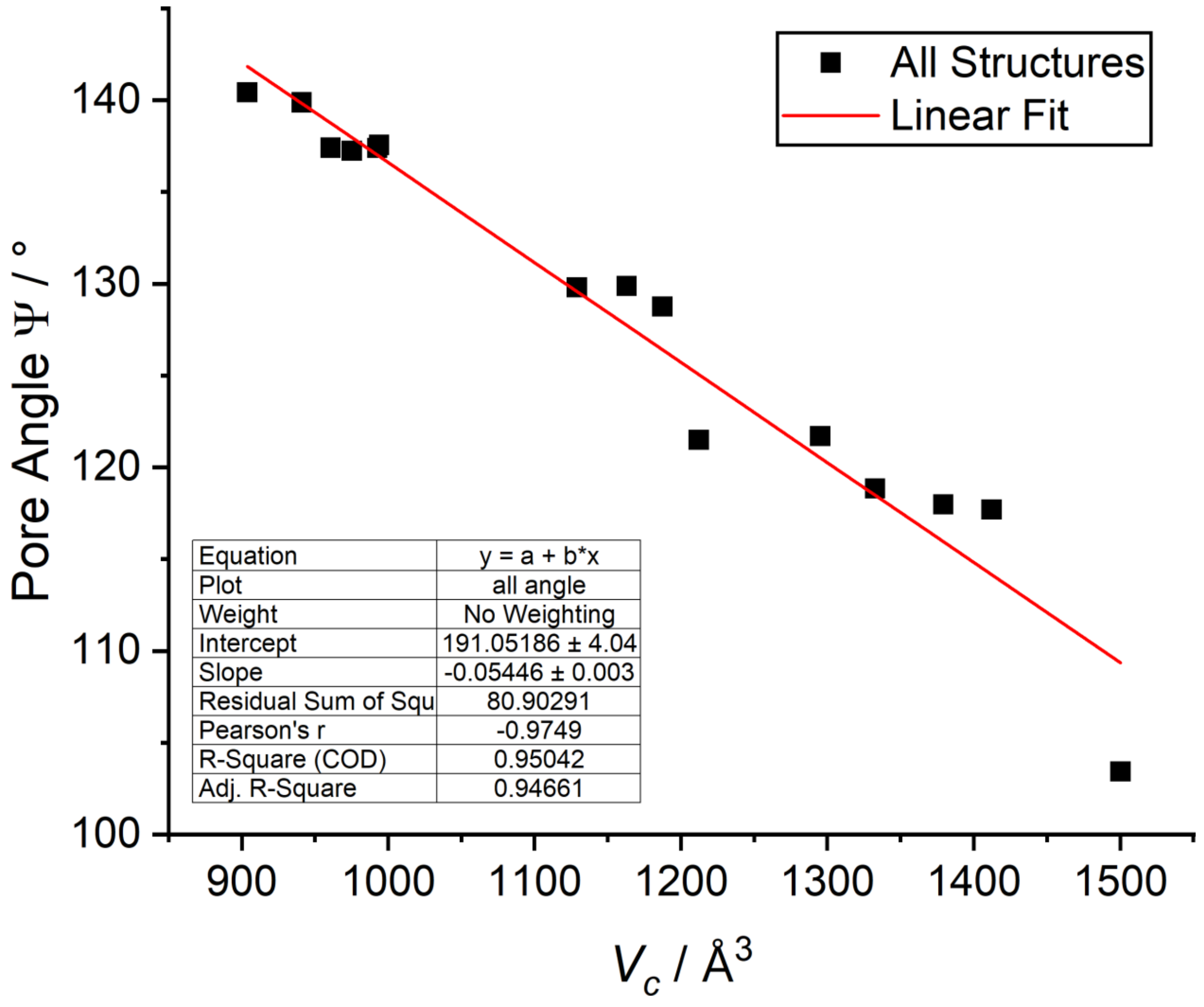

Figure S14. Plot of pore angle Ψ against corrected unit cell volume V_c for all structures of MIL-53(Cr) and MIL-53(Ga) collected by electron diffraction, with a linear data fit.

S6. Crystallographic Data Tables

S6.1. MIL-53(Cr)

**Identification code**	**CrMIL53_175_975_exp_126**
Empirical formula	$C_8H_{6.71}CrO_{5.85}$
Formula weight	248.477
Temperature / K	175
Crystal system	monoclinic
Space group	*I*2/*a*
a / Å	6.7892(16)
b / Å	7.517(5)
c / Å	19.202(4)
α / °	90
β / °	95.80(2)
γ / °	90
Volume / Å^3	975.0(7)
Z	4
ρ_{calc} g/cm^3	1.693
M / mm^{-1}	0.000
F(000)	168.7
Crystal size / mm^3	0.00125 × 0.0006 × 0.0001
Radiation	electron (λ = 0.02510)
2Θ range for data collection / °	0.2 to 1.8
Index ranges	-8 ≤ h ≤ 8, -8 ≤ k ≤ 8, -23 ≤ l ≤ 23
Reflections collected	1921
Independent reflections	719 [R_{int} = 0.0770, R_{sigma} = 0.1172]
Data/restraints/parameters	719/1/79
Goodness-of-fit on F^2	1.674
Final R indexes [I>=2σ (I)]	R_1 = 0.1664, wR_2 = 0.4114
Final R indexes [all data]	R_1 = 0.2013, wR_2 = 0.4227
Largest diff. peak/hole / e Å^{-3}	0.99/-0.69

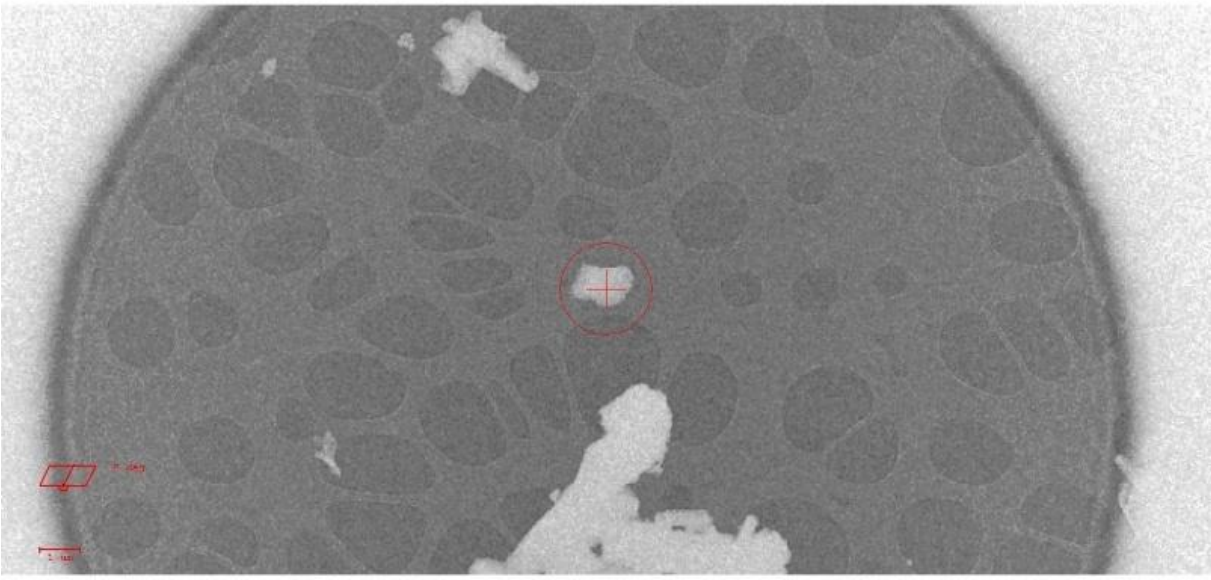

Figure S15. Electron microscopy image of the crystallite used to collect dataset CrMIL53_175_975_exp_126. Scale bar 1 μm.

**Identification code**	**CrMIL53_175_1985_exp116**
Empirical formula	$C_8H_7CrO_6$
Formula weight	251.134
Temperature / K	175
Crystal system	monoclinic
Space group	$P2_1/c$
a / Å	6.8366(10)
b / Å	15.103(3)
c / Å	19.3338(14)
α / °	90
β / °	96.039(11)
γ / °	90
Volume / $Å^3$	1985.2(5)
Z	8
$ρ_{calc}$ g/cm^3	1.681
M / mm^{-1}	0.000
F(000)	341.0
Crystal size / mm^3	0.00175 × 0.00125 × 0.0001
Radiation	electron (λ = 0.0251)
2Θ range for data collection / °	0.18 to 1.8
Index ranges	-7 ≤ h ≤ 7, -14 ≤ k ≤ 14, -24 ≤ l ≤ 24
Reflections collected	6373
Independent reflections	1800 [R_{int} = 0.0962, R_{sigma} = 0.1015]
Data/restraints/parameters	1800/425/286
Goodness-of-fit on F^2	1.447
Final R indexes [I>=2σ (I)]	R_1 = 0.1404, wR_2 = 0.3962
Final R indexes [all data]	R_1 = 0.1762, wR_2 = 0.4190
Largest diff. peak/hole / e $Å^{-3}$	0.61/-0.56

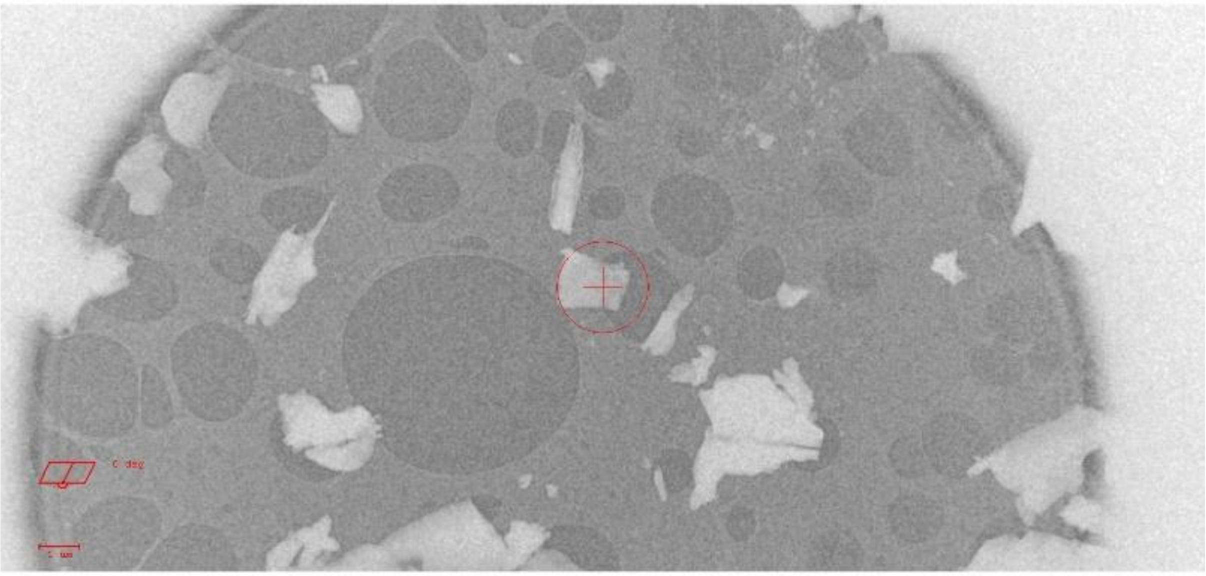

Figure S16. Electron microscopy image of the crystallite used to collect dataset CrMIL53_175_1985_exp116. Scale bar 1 μm.

**Identification code**	**CrMIL53_100_1260_exp_147**
Empirical formula	$C_8H_5CrO_5$
Formula weight	233.119
Temperature / K	100
Crystal system	orthorhombic
Space group	*Imma*
a / Å	18.254(14)
b / Å	6.890(4)
c / Å	10.97(3)
α / °	90
β / °	90
γ / °	90
Volume / $Å^3$	1379(4)
Z	4
ρ_{calc} g/cm^3	1.123
M / mm^{-1}	0.000
F(000)	158.3
Crystal size / mm^3	0.001 × 0.0007 × 0.0001
Radiation	electron (λ = 0.0251)
2Θ range for data collection / °	0.16 to 1.8
Index ranges	-22 ≤ h ≤ 22, -8 ≤ k ≤ 8, -13 ≤ l ≤ 13
Reflections collected	3192
Independent reflections	734 [R_{int} = 0.1664, R_{sigma} = 0.1488]
Data/restraints/parameters	734/1/42
Goodness-of-fit on F^2	1.637
Final R indexes [I>=2σ (I)]	R_1 = 0.2070, wR_2 = 0.4810
Final R indexes [all data]	R_1 = 0.2831, wR_2 = 0.5081
Largest diff. peak/hole / e $Å^{-3}$	0.88/-0.71

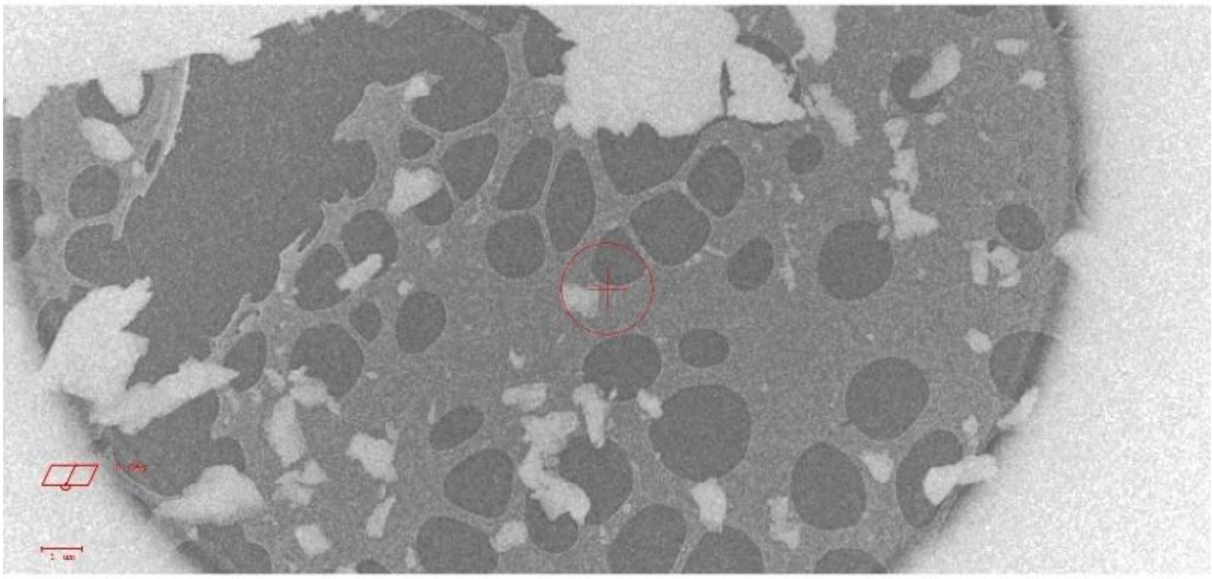

Figure S17. Electron microscopy image of the crystallite used to collect dataset CrMIL53_100_1260_exp_147. Scale bar 1 μm.

**Identification code**	**CrMIL53_240_1300_exp_132**
Empirical formula	$C_8H_5CrO_5$
Formula weight	233.119
Temperature / K	240
Crystal system	orthorhombic
Space group	*Imma*
a / Å	18.449(19)
b / Å	6.821(3)
c / Å	10.29(3)
α / °	90
β / °	90
γ / °	90
Volume / Å^3	1295(5)
Z	4
ρ_{calc} g/cm^3	1.196
M / mm^{-1}	0.000
F(000)	158.3
Crystal size / mm^3	0.00055 × 0.00045 × 0.0001
Radiation	electron (λ = 0.0251)
2Θ range for data collection / °	0.16 to 1.8
Index ranges	-22 ≤ h ≤ 22, -8 ≤ k ≤ 8, -12 ≤ l ≤ 12
Reflections collected	2712
Independent reflections	693 [R_{int} = 0.1372, R_{sigma} = 0.1457]
Data/restraints/parameters	693/7/41
Goodness-of-fit on F^2	1.318
Final R indexes [I>=2σ (I)]	R_1 = 0.1692, wR_2 = 0.4215
Final R indexes [all data]	R_1 = 0.2354, wR_2 = 0.4503
Largest diff. peak/hole / e Å^{-3}	0.99/-0.60

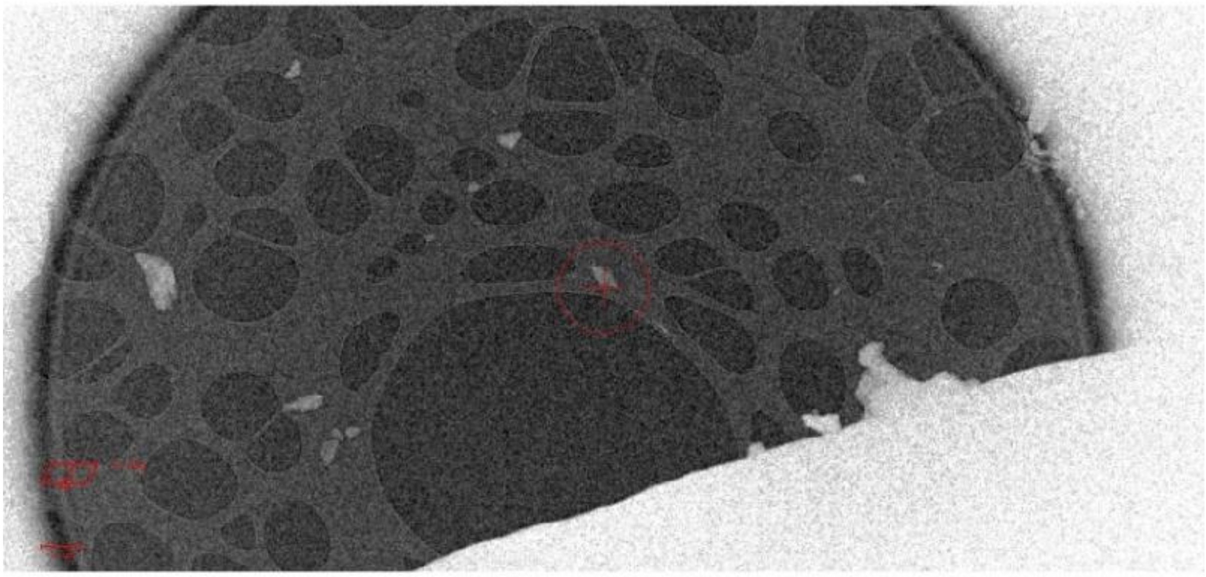

Figure S18. Electron microscopy image of the crystallite used to collect dataset CrMIL53_240_1300_exp_132. Scale bar 1 μm.

**Identification code**	**CrMIL53_300_1330_exp_136**
Empirical formula	$C_8H_5CrO_5$
Formula weight	233.119
Temperature / K	300
Crystal system	orthorhombic
Space group	*Imma*
a / Å	6.8514(12)
b / Å	18.143(9)
c / Å	10.719(9)
α / °	90
β / °	90
γ / °	90
Volume / $Å^3$	1332.4(13)
Z	4
ρ_{calc} g/cm^3	1.162
M / mm^{-1}	0.000
F(000)	158.3
Crystal size / mm^3	0.001 × 0.0003 × 0.0001
Radiation	electron (λ = 0.0251)
2Θ range for data collection / °	0.28 to 1.8
Index ranges	-8 ≤ h ≤ 8, -22 ≤ k ≤ 22, -13 ≤ l ≤ 13
Reflections collected	2724
Independent reflections	669 [R_{int} = 0.2369, R_{sigma} = 0.2878]
Data/restraints/parameters	669/43/42
Goodness-of-fit on F^2	1.393
Final R indexes [I>=2σ (I)]	R_1 = 0.1760, wR_2 = 0.4097
Final R indexes [all data]	R_1 = 0.2636, wR_2 = 0.4390
Largest diff. peak/hole / e $Å^{-3}$	0.98/-0.59

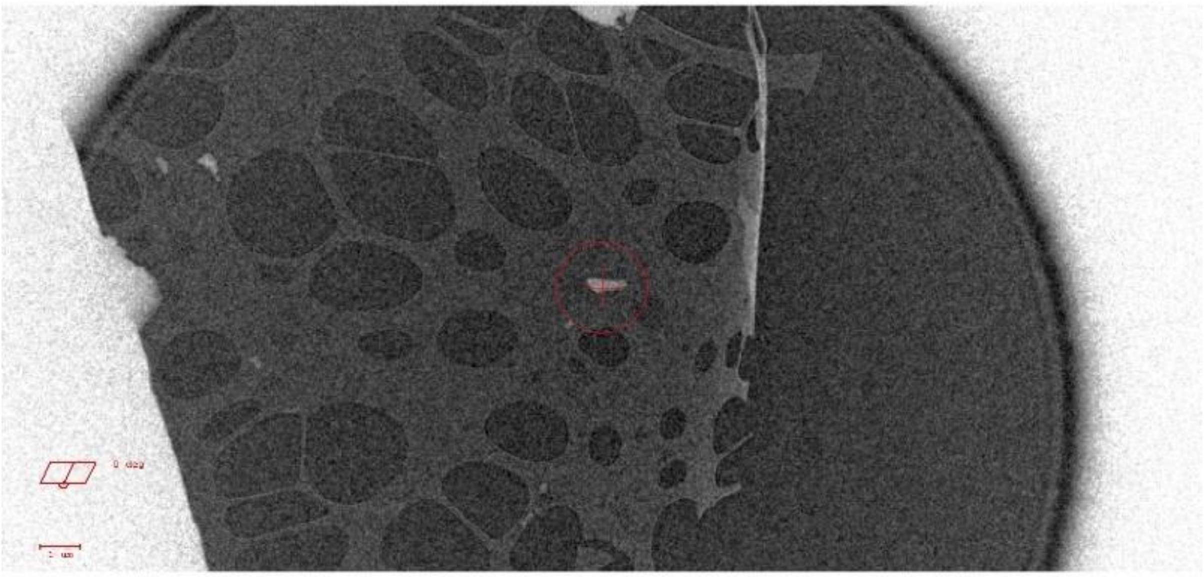

Figure S19. Electron microscopy image of the crystallite used to collect dataset CrMIL53_300_1330_exp_136. Scale bar 1 μm.

**Identification code**	**CrMIL53_300_1410_exp_138**
Empirical formula	$C_8H_5CrO_5$
Formula weight	233.119
Temperature / K	300
Crystal system	orthorhombic
Space group	*Imma*
a / Å	18.347(11)
b / Å	6.941(2)
c / Å	11.088(17)
α / °	90
β / °	90
γ / °	90
Volume / Å^3	1412(2)
Z	4
ρ_{calc} g/cm^3	1.097
M / mm^{-1}	0.000
F(000)	158.3
Crystal size / mm^3	0.00095 × 0.00035 × 0.0001
Radiation	electron (λ = 0.0251)
2Θ range for data collection / °	0.26 to 1.8
Index ranges	-22 ≤ h ≤ 22, -8 ≤ k ≤ 8, -12 ≤ l ≤ 11
Reflections collected	2519
Independent reflections	644 [R_{int} = 0.1472, R_{sigma} = 0.1599]
Data/restraints/parameters	644/1/41
Goodness-of-fit on F^2	1.405
Final R indexes [I>=2σ (I)]	R_1 = 0.1756, wR_2 = 0.4155
Final R indexes [all data]	R_1 = 0.2429, wR_2 = 0.4444
Largest diff. peak/hole / e Å^{-3}	0.87/-0.92

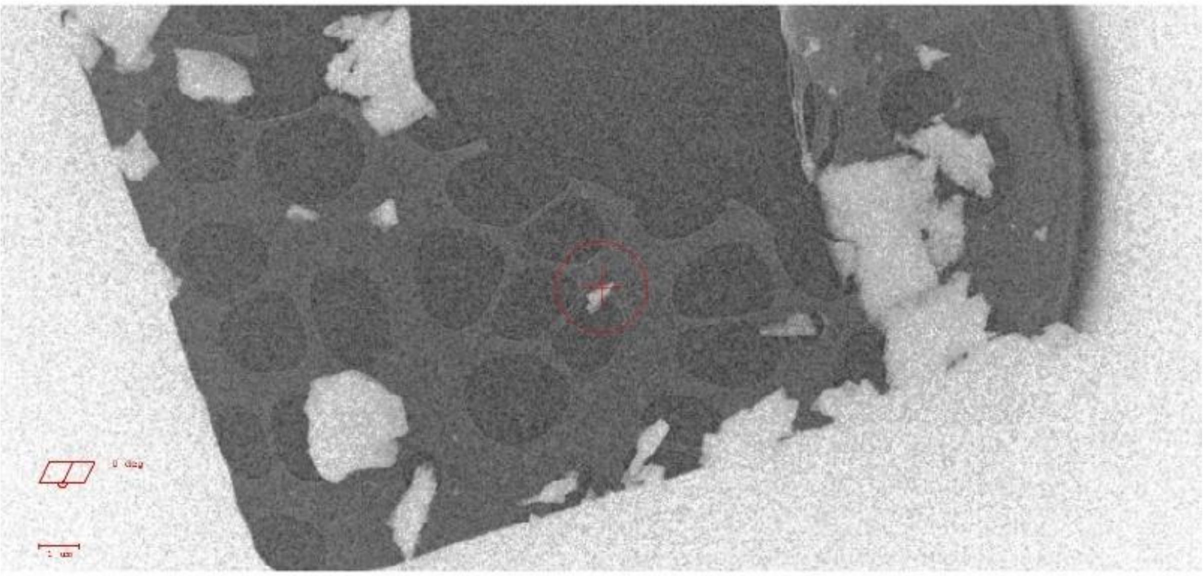

Figure S20. Electron microscopy image of the crystallite used to collect dataset CrMIL53_300_1410_exp_138. Scale bar 1 μm.

**Identification code**	**CrMIL53_RT_1500_exp_118**
Empirical formula	$C_8H_5CrO_5$
Formula weight	233.119
Temperature / K	298
Crystal system	orthorhombic
Space group	*Imma*
a / Å	6.8369(12)
b / Å	16.674(6)
c / Å	13.159(9)
α / °	90
β / °	90
γ / °	90
Volume / Å^3	1500.1(12)
Z	4
ρ_{calc} g/cm^3	1.032
M / mm^{-1}	0.000
F(000)	158.3
Crystal size / mm^3	0.00045 × 0.00035 × 0.0001
Radiation	electron (λ = 0.0251)
2Θ range for data collection / °	0.14 to 1.8
Index ranges	-8 ≤ h ≤ 8, -20 ≤ k ≤ 20, -13 ≤ l ≤ 14
Reflections collected	3175
Independent reflections	591 [R_{int} = 0.1490, R_{sigma} = 0.0938]
Data/restraints/parameters	591/1/41
Goodness-of-fit on F^2	1.533
Final R indexes [I>=2σ (I)]	R_1 = 0.1548, wR_2 = 0.4039
Final R indexes [all data]	R_1 = 0.1776, wR_2 = 0.4183
Largest diff. peak/hole / e Å^{-3}	0.44/-0.68

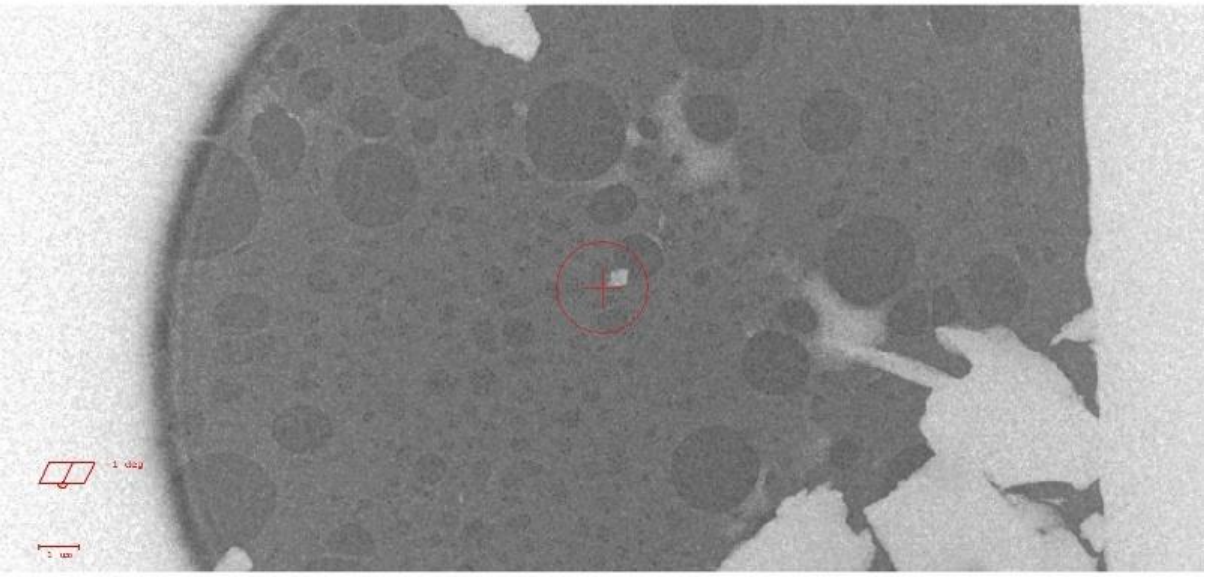

Figure S21. Electron microscopy image of the crystallite used to collect dataset CrMIL53_RT_1500_exp_118. Scale bar 1 μm.

S6.2. MIL-53(Ga)

**Identification code**	**GaMIL53_RT_900_Exp_384**
Empirical formula	$C_8H_5GaO_5$
Formula weight	250.85
Temperature / K	298
Crystal system	monoclinic
Space group	*I*2/*a*
a / Å	6.7449(19)
b / Å	6.959(8)
c / Å	19.343(6)
α / °	90
β / °	95.57(3)
γ / °	90
Volume / Å^3	903.7(12)
Z	4
ρ_{calc} g/cm^3	1.844
M / mm^{-1}	0.000
F(000)	159.0
Crystal size / mm^3	0.00075 × 0.00045 × 0.0001
Radiation	electron (λ = 0.0251)
2Θ range for data collection / °	0.324 to 1.8
Index ranges	-8 ≤ h ≤ 8, -8 ≤ k ≤ 8, -23 ≤ l ≤ 23
Reflections collected	2372
Independent reflections	715 [R_{int} = 0.1733, R_{sigma} = 0.1725]
Data/restraints/parameters	715/1/69
Goodness-of-fit on F^2	1.147
Final R indexes [I>=2σ (I)]	R_1 = 0.1390, wR_2 = 0.3610
Final R indexes [all data]	R_1 = 0.2097, wR_2 = 0.4013
Largest diff. peak/hole / e Å^{-3}	0.12/-0.13

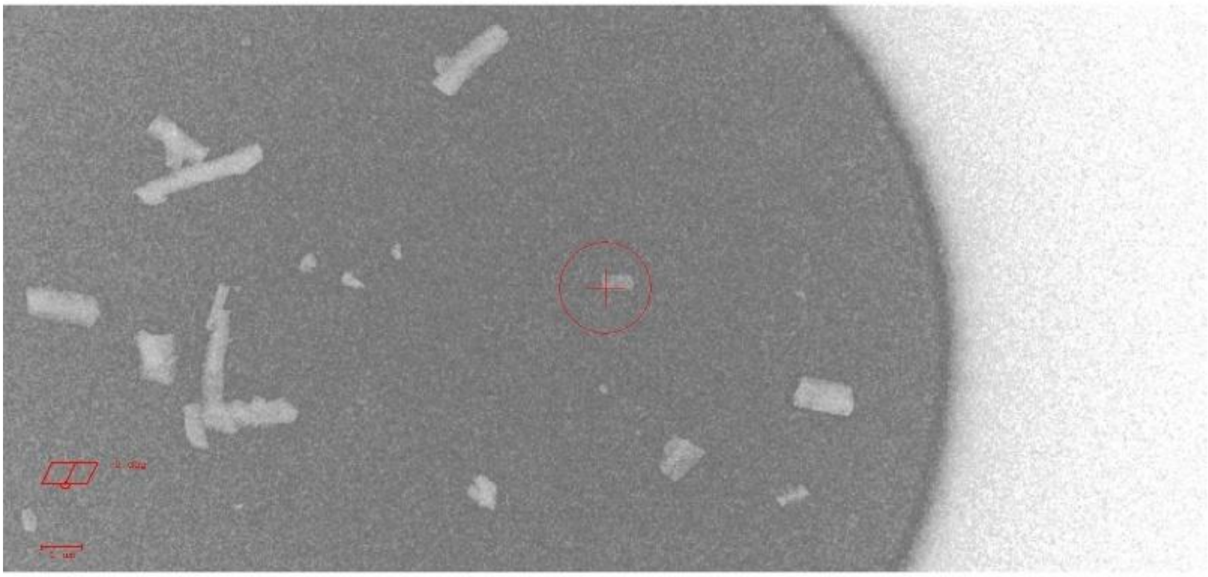

Figure S22. Electron microscopy image of the crystallite used to collect dataset GaMIL53_RT_900_Exp_384. Scale bar 1 µm.

**Identification code**	**GaMIL53_327_940_Exp_402**
Empirical formula	$C_8H_5GaO_5$
Formula weight	250.85
Temperature / K	327
Crystal system	monoclinic
Space group	*I*2/*a*
a / Å	6.7570(12)
b / Å	7.145(6)
c / Å	19.569(3)
α / °	90
β / °	95.266(13)
γ / °	90
Volume / Å^3	940.8(8)
Z	4
ρ_{calc} g/cm^3	1.771
M / mm^{-1}	0.000
F(000)	159.0
Crystal size / mm^3	0.00225 × 0.00085 × 0.0001
Radiation	electron (λ = 0.0251)
2Θ range for data collection / °	0.148 to 1.798
Index ranges	-8 ≤ h ≤ 8, -8 ≤ k ≤ 8, -24 ≤ l ≤ 24
Reflections collected	1995
Independent reflections	732 [R_{int} = 0.0699, R_{sigma} = 0.0903]
Data/restraints/parameters	732/67/69
Goodness-of-fit on F^2	1.649
Final R indexes [I>=2σ (I)]	R_1 = 0.1528, wR_2 = 0.4291
Final R indexes [all data]	R_1 = 0.1893, wR_2 = 0.4433
Largest diff. peak/hole / e Å^{-3}	0.20/-0.1

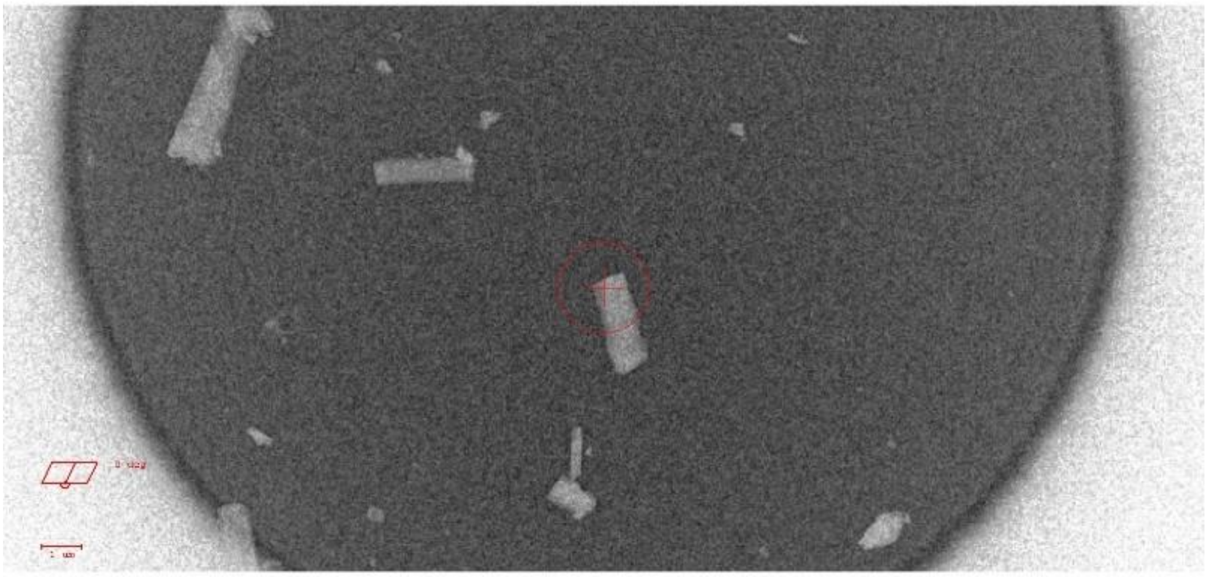

Figure S23. Electron microscopy image of the crystallite used to collect dataset GaMIL53_327_940_Exp_402. Scale bar 1 µm.

**Identification code**	**GaMIL53_175_960_Exp_387**
Empirical formula	$C_8H_7GaO_6$
Formula weight	268.86
Temperature / K	175
Crystal system	monoclinic
Space group	*I*2/*a*
a / Å	6.6980(18)
b / Å	7.498(6)
c / Å	19.239(5)
α / °	90
β / °	96.20(3)
γ / °	90
Volume / Å^3	960.5(8)
Z	4
ρ_{calc} g/cm^3	1.859
M / mm^{-1}	0.000
F(000)	171.0
Crystal size / mm^3	0.00085 × 0.00075 × 0.0001
Radiation	electron (λ = 0.0251)
2Θ range for data collection / °	0.15 to 1.794
Index ranges	-8 ≤ h ≤ 8, -7 ≤ k ≤ 7, -23 ≤ l ≤ 23
Reflections collected	1829
Independent reflections	598 [R_{int} = 0.0836, R_{sigma} = 0.0854]
Data/restraints/parameters	598/1/76
Goodness-of-fit on F^2	1.213
Final R indexes [I>=2σ (I)]	R_1 = 0.1288, wR_2 = 0.3393
Final R indexes [all data]	R_1 = 0.1458, wR_2 = 0.3502
Largest diff. peak/hole / e Å^{-3}	0.14/-0.16

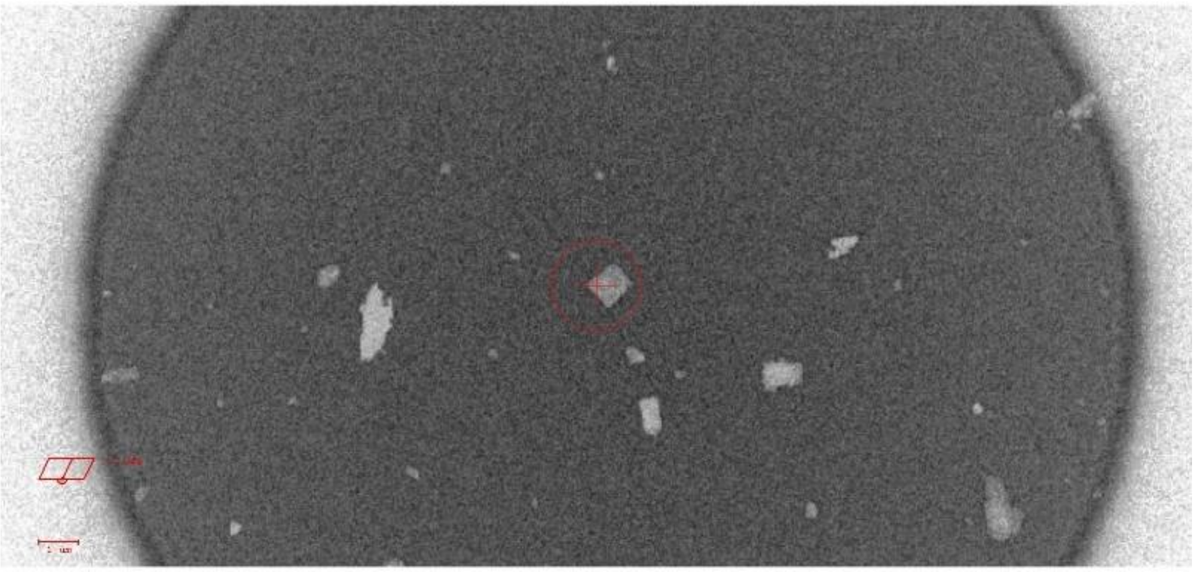

Figure S24. Electron microscopy image of the crystallite used to collect dataset GaMIL53_175_960_Exp_387. Scale bar 1 µm.

**Identification code**	**GaMIL53_175_1990_Exp_392**
Empirical formula	$C_8H_7GaO_6$
Formula weight	268.861
Temperature / K	175
Crystal system	monoclinic
Space group	$P2_1/c$
a / Å	6.7487(3)
b / Å	15.1816(18)
c / Å	19.5011(10)
α / °	90
β / °	95.922(5)
γ / °	90
Volume / Å^3	1987.3(3)
Z	8
ρ_{calc} g/cm^3	1.797
M / mm^{-1}	0.000
F(000)	342.1
Crystal size / mm^3	0.00225 × 0.00085 × 0.0001
Radiation	electron (λ = 0.02510)
2Θ range for data collection / °	0.12 to 1.8
Index ranges	-8 ≤ h ≤ 8, -17 ≤ k ≤ 17, -24 ≤ l ≤ 24
Reflections collected	8649
Independent reflections	3101 [R_{int} = 0.0904, R_{sigma} = 0.1291]
Data/restraints/parameters	3101/445/296
Goodness-of-fit on F^2	1.055
Final R indexes [I>=2σ (I)]	R_1 = 0.1417, wR_2 = 0.3428
Final R indexes [all data]	R_1 = 0.1692, wR_2 = 0.3598
Largest diff. peak/hole / e Å^{-3}	0.90/-1.10

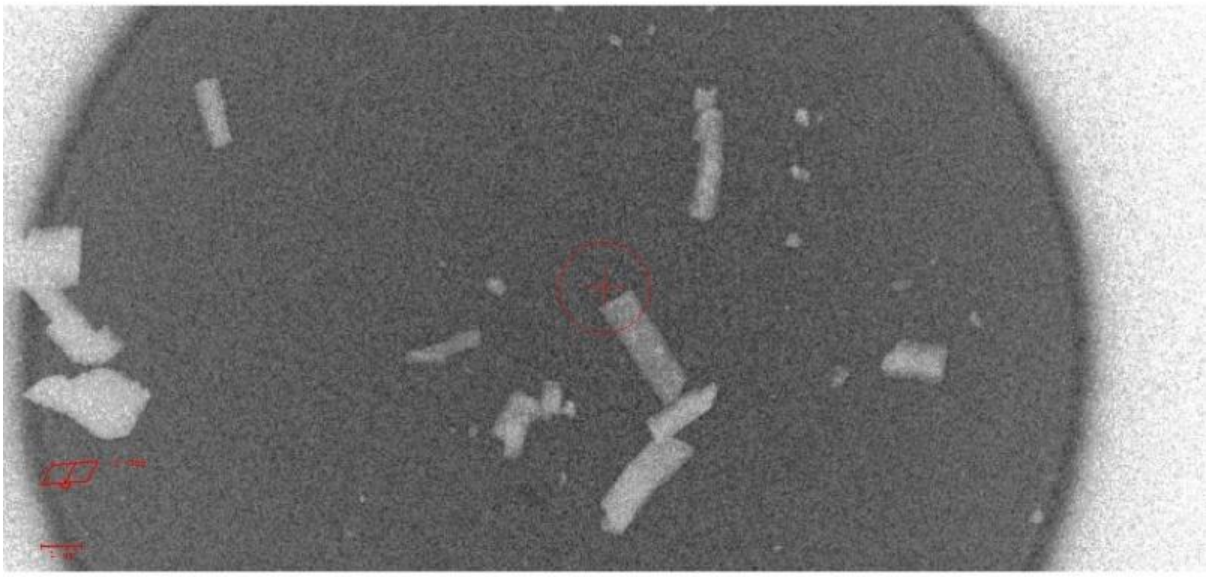

Figure S25. Electron microscopy image of the crystallite used to collect dataset GaMIL53_175_1990_Exp_392. Scale bar 1 μm.

Identification code	GaMIL53_175_1130_Exp_390
Empirical formula	$C_8H_{5.92}GaO_{5.46}$
Formula weight	259.14
Temperature / K	175
Crystal system	monoclinic
Space group	*I*2/*a*
a / Å	6.6980(6)
b / Å	8.8946(13)
c / Å	18.9895(14)
α / °	90
β / °	93.837(6)
γ / °	90
Volume / Å^3	1128.8(2)
Z	4
ρ_{calc} g/cm^3	1.525
M / mm^{-1}	0.000
F(000)	164.0
Crystal size / mm^3	0.00075 × 0.00045 × 0.0001
Radiation	electron (λ = 0.0251)
2Θ range for data collection / °	0.302 to 1.798
Index ranges	-7 ≤ h ≤ 7, -10 ≤ k ≤ 10, -23 ≤ l ≤ 23
Reflections collected	2688
Independent reflections	906 [R_{int} = 0.0830, R_{sigma} = 0.0860]
Data/restraints/parameters	906/76/73
Goodness-of-fit on F^2	1.189
Final R indexes [I>=2σ (I)]	R_1 = 0.1221, wR_2 = 0.3108
Final R indexes [all data]	R_1 = 0.1395, wR_2 = 0.3198
Largest diff. peak/hole / e Å^{-3}	0.22/-0.13

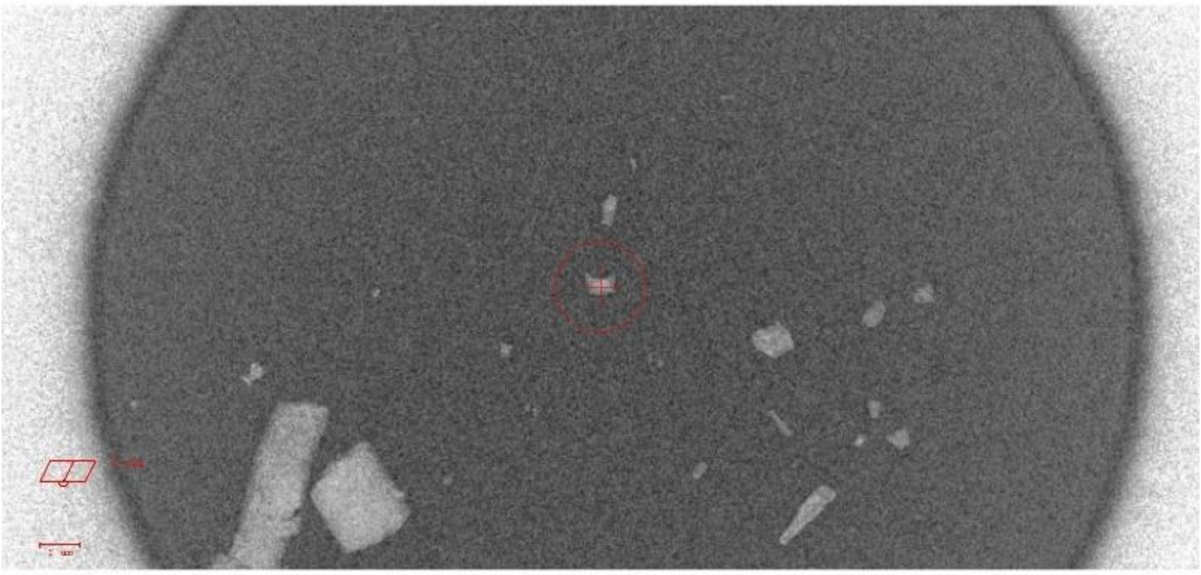

Figure S26. Electron microscopy image of the crystallite used to collect dataset GaMIL53_175_1130_Exp_390. Scale bar 1 μm.

**Identification code**	**GaMIL53_100_1160_Exp_409**
Empirical formula	$C_8H_{5.6}GaO_{5.3}$
Formula weight	256.25
Temperature / K	100
Crystal system	monoclinic
Space group	*I*2/*a*
a / Å	6.7860(7)
b / Å	8.962(3)
c / Å	19.167(2)
α / °	90
β / °	94.111(10)
γ / °	90
Volume / $Å^3$	1162.7(4)
Z	4
ρ_{calc} g/cm^3	1.464
M / mm^{-1}	0.000
F(000)	163.0
Crystal size / mm^3	0.00075 × 0.0005 × 0.0001
Radiation	electron (λ = 0.0251)
2Θ range for data collection / °	0.266 to 1.798
Index ranges	-8 ≤ h ≤ 8, -8 ≤ k ≤ 9, -23 ≤ l ≤ 23
Reflections collected	2351
Independent reflections	764 [R_{int} = 0.0759, R_{sigma} = 0.0743]
Data/restraints/parameters	764/45/73
Goodness-of-fit on F^2	1.185
Final R indexes [I>=2σ (I)]	R_1 = 0.1102, wR_2 = 0.2749
Final R indexes [all data]	R_1 = 0.1274, wR_2 = 0.2847
Largest diff. peak/hole / e Å^{-3}	0.13/-0.11

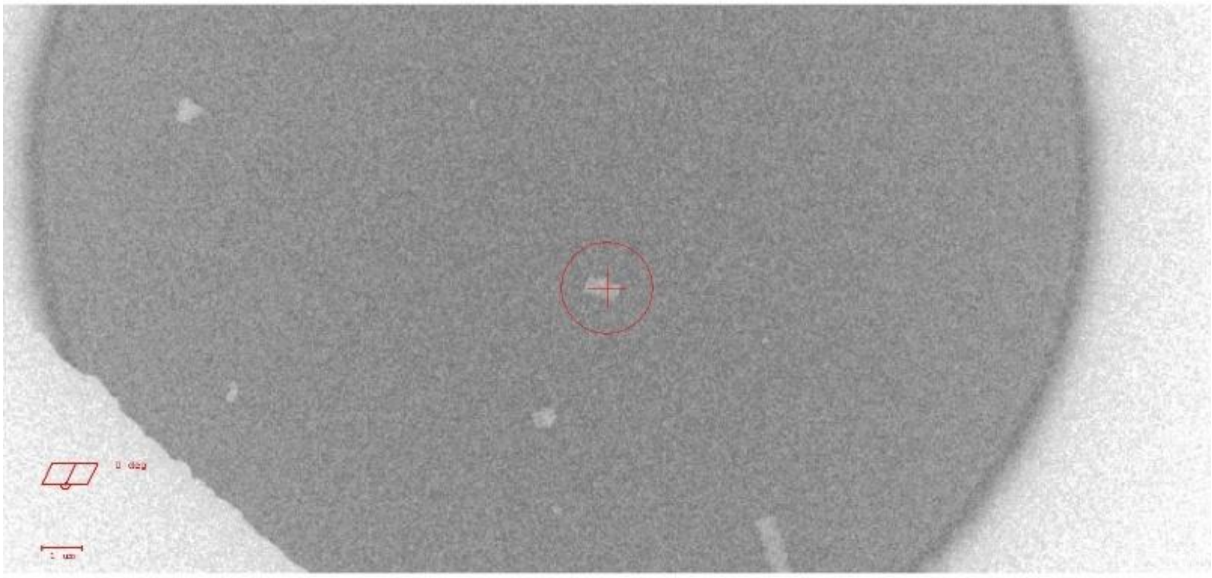

Figure S27. Electron microscopy image of the crystallite used to collect dataset GaMIL53_100_1160_Exp_409. Scale bar 1 μm.

**Identification code**	**GaMIL53_327_1190_Exp_403**
Empirical formula	$C_8H_{5.8}GaO_{5.4}$
Formula weight	258.05
Temperature / K	327
Crystal system	monoclinic
Space group	*I*2/*a*
a / Å	6.7672(7)
b / Å	9.179(2)
c / Å	19.1391(18)
α / °	90
β / °	93.136(8)
γ / °	90
Volume / Å^3	1187.1(4)
Z	4
ρ_{calc} g/cm^3	1.444
M / mm^{-1}	0.000
F(000)	164.0
Crystal size / mm^3	0.00075 × 0.0004 × 0.0001
Radiation	electron (λ = 0.0251)
2Θ range for data collection / °	0.298 to 1.8
Index ranges	-7 ≤ h ≤ 7, -10 ≤ k ≤ 10, -23 ≤ l ≤ 23
Reflections collected	2912
Independent reflections	954 [R_{int} = 0.1019, R_{sigma} = 0.1151]
Data/restraints/parameters	954/0/75
Goodness-of-fit on F^2	1.165
Final R indexes [I>=2σ (I)]	R_1 = 0.1093, wR_2 = 0.2936
Final R indexes [all data]	R_1 = 0.1379, wR_2 = 0.3090
Largest diff. peak/hole / e Å^{-3}	0.12/-0.17

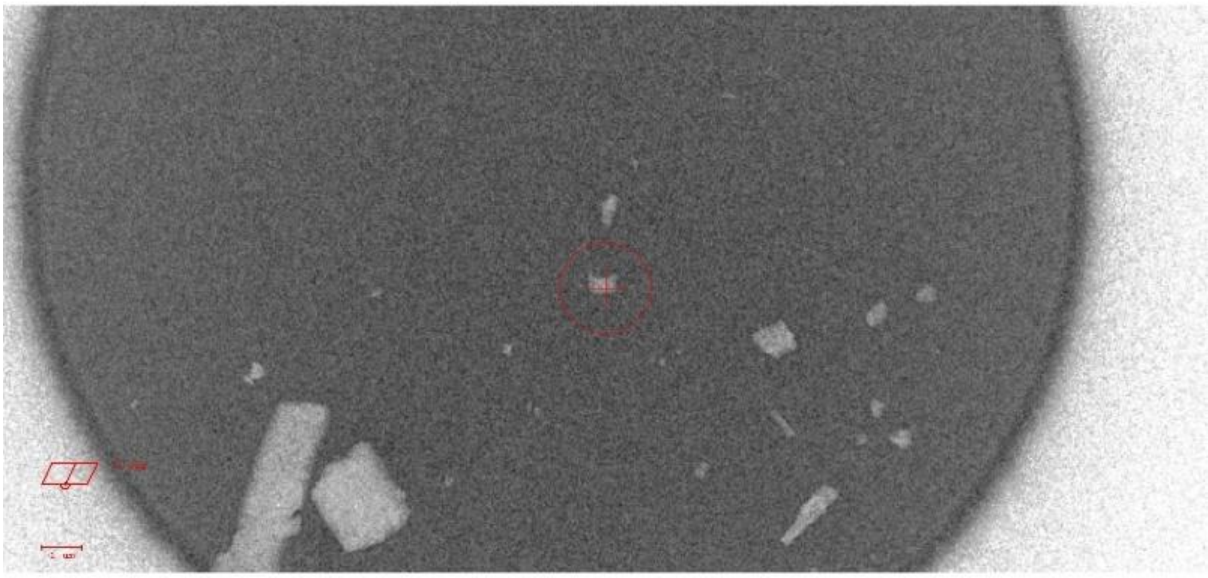

Figure S28. Electron microscopy image of the crystallite used to collect dataset GaMIL53_327_1190_Exp_403. Scale bar 1 μm.

**Identification code**	**GaMIL53_100_1300_Exp_408**
Empirical formula	$C_8H_{5.8}GaO_{5.4}$
Formula weight	258.052
Temperature / K	100
Crystal system	monoclinic
Space group	*I*2/*a*
a / Å	6.632(5)
b / Å	10.12(2)
c / Å	18.07(6)
α / °	90
β / °	92.31(11)
γ / °	90
Volume / Å^3	1212(5)
Z	4
ρ_{calc} g/cm^3	1.414
Μ / mm^{-1}	0.000
F(000)	163.7
Crystal size / mm^3	0.0012 × 0.0004 × 0.0001
Radiation	electron (λ = 0.02510)
2Θ range for data collection / °	0.16 to 1.86
Index ranges	-7 ≤ h ≤ 7, -12 ≤ k ≤ 12, -23 ≤ l ≤ 23
Reflections collected	2905
Independent reflections	1229 [R_{int} = 0.0963, R_{sigma} = 0.1298]
Data/restraints/parameters	1229/94/73
Goodness-of-fit on F^2	1.563
Final R indexes [I>=2σ (I)]	R_1 = 0.1882, wR_2 = 0.4501
Final R indexes [all data]	R_1 = 0.2502, wR_2 = 0.4753
Largest diff. peak/hole / e Å^{-3}	1.58/-1.04

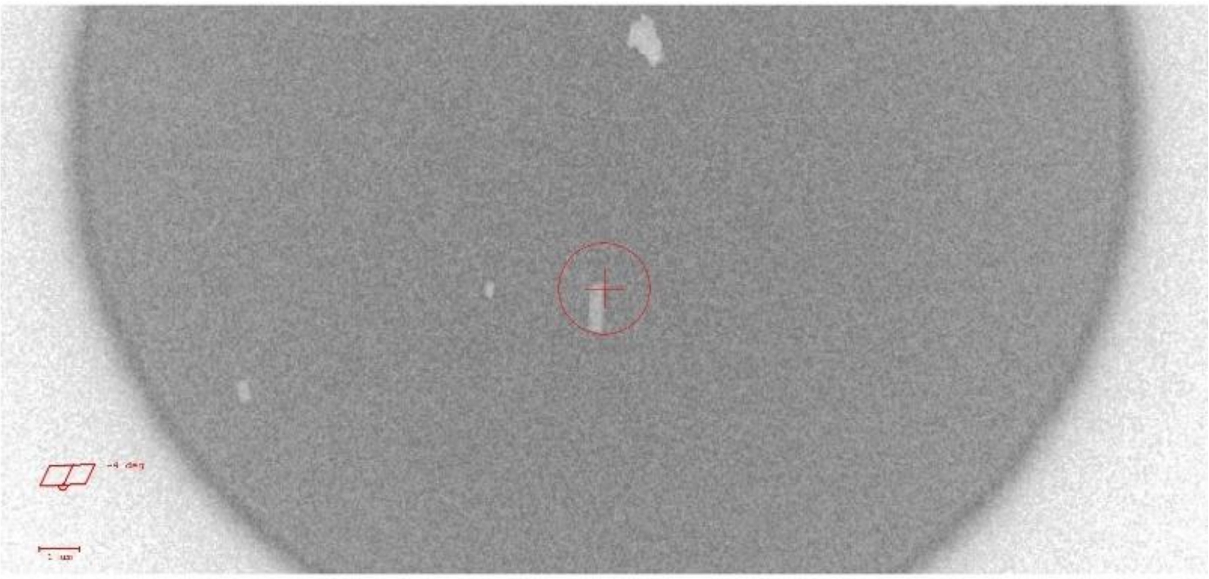

Figure S29. Electron microscopy image of the crystallite used to collect dataset GaMIL53_100_1300_Exp_408. Scale bar 1 μm.

S7. References

[S1] V. Petříček, L. Palatinus, J. Plášil, M. Dušek, *Z. Kristallogr. Cryst. Mater.* **2023**, *238*, 271-282.

[S2] S. Ito, F. J. White, E. Okunishi, Y. Aoyama, A. Yamano, H. Sato, J. D. Ferrara, M. Jasnowski, M. Meyer, *CrystEngComm* **2021**, *23*, 8622-8630.

[S3] Rigaku Corporation, CrysAlisPro ver. 171.44.81a, Rigaku Oxford Diffraction Ltd, Wroclaw, Poland, **2024**.

[S4] G. Sheldrick, *Acta Crystallogr. Sect. A* **2015**, *71*, 3-8.

[S5] G. Sheldrick, *Acta Crystallogr. Sect. C* **2015**, *71*, 3-8.

[S6] L. J. Bourhis, O. V. Dolomanov, R. J. Gildea, J. A. K. Howard, H. Puschmann, *Acta Crystallog. Sect. A* **2015**, *71*, 59-75.

[S7] L. M. Peng, *Micron* **1999**, *30*, 625-648.

[S8] F. H. Allen, I. J. Bruno, *Acta Crystallogr. Sect. B* **2010**, *66*, 380-386.

[S9] V. B. López-Cervantes, D. Bara, A. Yañez-Aulestia, E. Martínez-Ahumada, A. López-Olvera, Y. A. Amador-Sánchez, D. Solis-Ibarra, E. Sánchez-González, I. A. Ibarra, R. S. Forgan, *Chem. Commun.* **2023**, *59*, 8115-8118.

[S10] C. Serre, F. Millange, C. Thouvenot, M. Noguès, G. Marsolier, D. Louër, G. Férey, *J. Am. Chem. Soc.* **2002**, *124*, 13519-13526.

[S11] N. Guillou, F. Millange, R. I. Walton, *Chem. Commun.* **2011**, *47*, 713-715.

[S12] F. Millange, N. Guillou, R. I. Walton, J.-M. Grenèche, I. Margiolaki, G. Férey, *Chem. Commun.* **2008**, 4732-4734.

[S13] F. Millange, N. Guillou, M. E. Medina, G. Férey, A. Carlin-Sinclair, K. M. Golden, R. I. Walton, *Chem. Mater.* **2010**, *22*, 4237-4245.

[S14] T. Loiseau, C. Serre, C. Huguenard, G. Fink, F. Taulelle, M. Henry, T. Bataille, G. Férey, *Chem. Eur. J.* **2004**, *10*, 1373-1382.

[S15] Y. Ling, T. Sun, L. Guo, X. Si, Y. Jiang, Q. Zhang, Z. Chen, O. Terasaki, Y. Ma, *Nat. Commun.* **2022**, *13*, 6625.

[S16] G. Ortiz, G. Chaplais, J.-L. Paillaud, H. Nouali, J. Patarin, J. Raya, C. Marichal, *J. Phys. Chem. C* **2014**, *118*, 22021-22029.

[S17] A. Boutin, D. Bousquet, A. U. Ortiz, F.-X. Coudert, A. H. Fuchs, A. Ballandras, G. Weber, I. Bezverkhyy, J.-P. Bellat, G. Ortiz, G. Chaplais, J.-L. Paillaud, C. Marichal, H. Nouali, J. Patarin, *J. Phys. Chem. C* **2013**, *117*, 8180-8188.

[S18] C. Volkringer, T. Loiseau, N. Guillou, G. Férey, E. Elkaïm, A. Vimont, *Dalton Trans.* **2009**, 2241-2249.

[S19] A. R. B. J. Lutton-Gething, L. T. Nangkam, J. O. W. Johansson, I. Pallikara, J. M. Skelton, G. F. S. Whitehead, I. Vitorica-Yrezabal, M. P. Attfield, *Chem. Eur. J.* **2023**, *29*, e202203773.

[S20] Y. Liu, J.-H. Her, A. Dailly, A. J. Ramirez-Cuesta, D. A. Neumann, C. M. Brown, *J. Am. Chem. Soc.* **2008**, *130*, 11813-11818.

[S21] J. P. S. Mowat, V. R. Seymour, J. M. Griffin, S. P. Thompson, A. M. Z. Slawin, D. Fairen-Jimenez, T. Düren, S. E. Ashbrook, P. A. Wright, *Dalton Trans.* **2012**, *41*, 3937-3941.
